# Orbitofrontal cortex contributes to context-dependent timing under ambiguity

**DOI:** 10.1016/j.isci.2026.117110

**Published:** 2026-08-10

**Authors:** Gabrielle Marmur, Haneen Rajabi, John Schwarcz, Robert Reiner, Jonathan Kadmon, Eran Lottem

**Affiliations:** 1 Edmond and Lily Safra Center for Brain Sciences (ELSC), The Hebrew University of Jerusalem, Jerusalem, Israel

**Keywords:** decision making, flexible timing, evidence accumulation, auditory change detection, orbitofrontal cortex, response timing

## Abstract

Adaptive decision-making requires flexibly adjusting the decision process when stimulus reliability changes. Most paradigms manipulate reliability by changing stimulus properties, whereas animals often face situations in which the physical inputs are unchanged but their informativeness varies. To address this, we designed a change-detection task in which the reward-predictive reliability of auditory cues varied across blocks while the cues themselves remained unchanged. Mice adjusted their waiting time accordingly, responding later when cues were less reliable. Importantly, these adjustments reflected sensitivity to the underlying sound statistics, rather than reliance solely on reward feedback. Inactivation of the orbitofrontal cortex (OFC) reduced this timing modulation. Modeling captured this effect as reduced context-dependent modulation of accumulation dynamics, accompanied by increased lapse-like responding. These findings suggest that OFC contributes to maintaining context-dependent timing under ambiguity and establish a tractable framework for studying flexible behavioral adjustment to changing cue statistics.

## Highlights

• Mice adapt response timing to context-dependent auditory statistics
• Behavioral adaptation reflects sensory statistics, not purely reward feedback
• Timing shifts are captured by modulation of drift rate in an evidence accumulation model
• OFC inactivation reduces structured timing and increases lapse-like responding

## Graphical abstract

**Figure:**
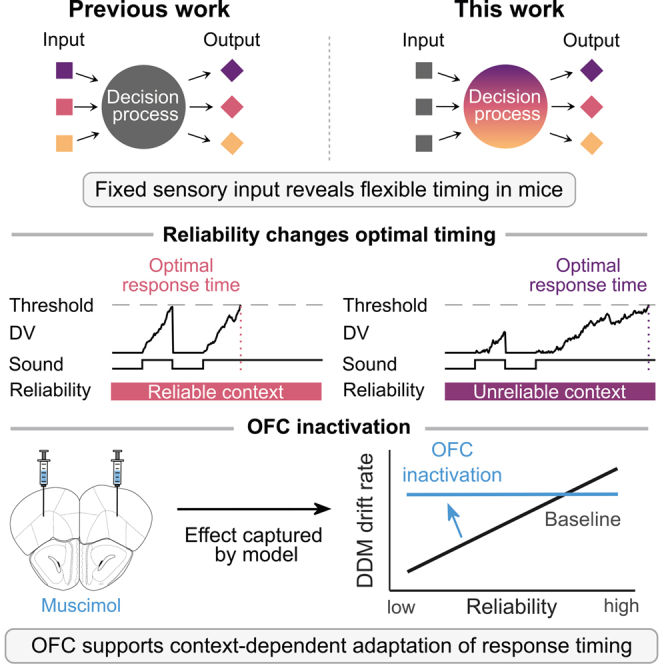

## Teaser

Biological sciences; Behavioral neuroscience; Biological sciences research methodologies

## Introduction

In Aesop’s fable of the boy who cried wolf, the villagers initially respond quickly to each cry. But after too many false alarms, they learn to ignore the untrustworthy shepherd boy. When inferring the hidden state of the environment (is there a wolf?) based on sensory cues (the shepherd boy’s cries), it is beneficial to adjust the inference process according to the reliability of those cues.

Normative models of decision making predict that less reliable evidence should be accumulated more cautiously, integrating over more evidence before reaching a decision.^1^^,^^2^ Drift-diffusion models (DDMs) formalize this process, with drift rate scaling with cue reliability.^3^^,^^4^^,^^5^^,^^6^ However, in most behavioral paradigms, stimulus reliability is manipulated by altering the sensory input itself (e.g., contrast,^7^ coherence,^8^^,^^9^ intensity^10^). This makes it difficult to determine how animals tune their decision computation based on an ongoing internal estimate of reliability when the physical stimulus is unchanged.

We address this gap with a novel auditory change-detection task in which mice need to infer latent state changes from ambiguous auditory cues. Critically, we manipulate the reliability of these cues not by altering the physical properties of the sound, but by changing how strongly each cue predicts a state change. This decouples stimulus features from their informativeness, forcing mice to adapt their accumulation strategy rather than simply apply fixed computations to different sensory inputs.

The ability to tune accumulation parameters based on cue reliability likely requires flexible, high-level representations of task structure. The orbitofrontal cortex (OFC) is well positioned for this role^11^: It encodes latent task states and supports behavior in partially observable environments.^12^^,^^13^^,^^14^ Yet, whether OFC is causally required for flexible control of evidence accumulation remains unknown.

Here, we combine behavioral modeling with reversible OFC inactivation to test this question. We fit a bounded evidence accumulation model to response-time (RT) distributions and show that mice adapt their accumulation rate to cue reliability. Silencing OFC selectively abolished this form of adaptation, revealing a causal role for OFC in tuning evidence accumulation based on an estimate of environmental reliability.

## Results

### An auditory change detection task with varying cue reliability

**Figure 1:**
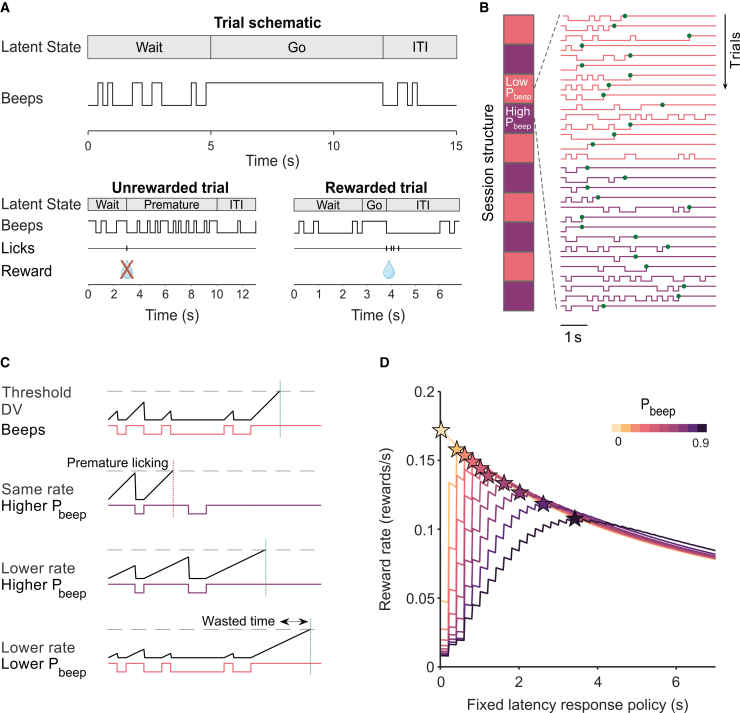
An auditory change-detection task with latent state structure (A) Trial structure. Each trial began in a “Wait” state in which beeps occurred with probability P_beep_. After a stochastic delay, if the mouse withheld licking, the environment transitioned to the “Go” state, where beeps occurred continuously. Licking during the “Wait” state triggered a 7 s timeout (bottom left - unrewarded trial), whereas licking during the “Go” state yielded a water reward and immediate transition to the inter-trial interval (ITI; bottom right - rewarded trial). (B) Session structure. Each row represents the auditory stimulus of one trial; P_beep_ varied in blocks of 30–50 trials (left column). Green dots indicate “Wait” to “Go” transitions. (C) Schematic of an optimal decision variable (DV) that ramps during beeps and resets during silences. In high-P_beep_ blocks, lower drift rates reduce premature licking, while in low-P_beep_ blocks, lower drift rates reduce reward rate. (D) Reward rate under fixed-latency policies. Optimal waiting times (stars) increase with P_beep_, formalizing the trade-off between premature responding and delayed rewards.

To investigate how animals adapt decision-making policies to changing evidence reliability, we developed a novel auditory-guided change-detection task with latent-state structure (Figure 1A). On each trial, head-fixed mice were presented with a sequence of auditory beeps and trained to lick a waterspout upon detecting an uncued transition from a latent “Wait” state of variable duration to a “Go” state. During “Wait,” each 0.2 s time bin contained an audible beep with probability P_beep_ or silence with probability 1-P_beep_; in “Go,” the beep was presented continuously. Licking during “Wait” was unrewarded and triggered a 7 s premature-lick timeout (Figure 1A), while licking during “Go” resulted in water delivery and an immediate transition to the inter-trial interval (ITI). In both the premature-lick timeout and the ITI, beeps continued probabilistically according to P_beep_; therefore, the sound stream provided no indication of trial boundary. Seeing as no explicit cue indicates the transition between “Wait” and “Go,” and the timing of this transition varies from trial to trial, the mice must detect the change point based solely on beep duration. The critical task parameter, P_beep_, governs the reliability of the reward-predictive cue, which is a sustained (continuous) beep. In the “Wait” state, a sustained beep appears when successive bins happen to contain beeps; the higher P_beep_ is, the more often such long beeps occur without a true state change. Consequently, as P_beep_ increases, the “Wait” sound stream more closely mimics the “Go” state, reducing the reliability of a specific beep duration. For intuition, at P_beep_ = 0.2, a 0.6 s beep (three consecutive 0.2 s bins) is highly informative that the transition has occurred and that it is safe to lick for reward (the chance it appears spuriously is ≈ 0.2^3^ ≈ 0.8%), whereas at P_beep_ = 0.8 the same 0.6 s beep is common (≈0.8^3^ ˜ 51%), making it a far less reliable cue for the current state.

To assess how mice adapt to these varying levels of cue reliability, we manipulated P_beep_ in blocks of 30–50 trials across a range of values (0–0.9), with each session containing multiple block transitions (Figure 1B). Because evidence is conveyed by beep duration, a simple way to achieve optimal behavior is to use a ramp-to-threshold decision variable (DV) that increases during beeps and resets during silence (Figure 1C). This follows from the optimal-policy analysis for our task,^15^ where the reward rate is maximized by a P_beep_-specific number of consecutive beeps before acting and where any silence collapses belief in the “Go” state to zero. In high P_beep_ blocks, fast accumulation risks premature licking and timeouts; in low P_beep_ blocks, slow accumulation reduces reward rate by delaying correct responses. To formalize this tradeoff, we calculated the expected reward rates for deterministic threshold-crossing policies (i.e., responding after a fixed beep duration) under each P_beep_ condition (Figure 1D, see Methods). These calculations show that the reward-rate-maximizing latency increases with P_beep_, providing normative predictions for how waiting times should adapt across contexts.

### Mice adapt response timing to cue reliability

**Figure 2:**
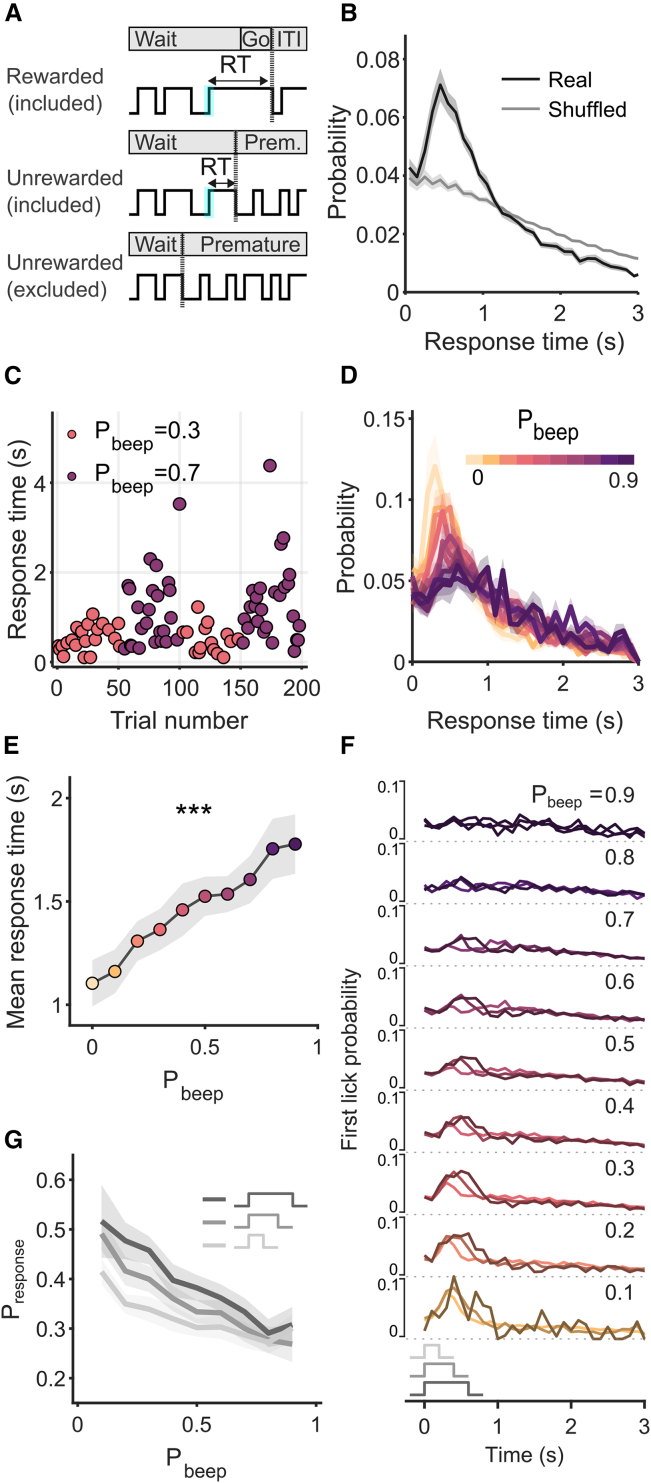
Mice wait longer when cue reliability is lower (A) Schematic of the response-time (RT) measure. On trials in which the mouse reached the onset of the beep that carried into the “Go” state, RT was defined as the time of the first lick relative to that beep onset (cyan), whether the lick was rewarded (top) or unrewarded (middle). Trials in which the mouse emitted a premature lick before reaching this beep were excluded. (B) Empirical RT distributions (0.1 s bins, mean ± SEM) differ from shuffled controls, indicating sound-driven timing. Data are represented as mean ± SEM across mice; 0.1 s bins; *n* = 15 mice. (C) Example session shows trial-by-trial response times across P_beep_ contexts; responses were delayed in higher P_beep_ blocks. (D) RT distributions for each context, showing systematic rightward shifts as P_beep_ increased. Data are represented as mean ± SEM across mice; 0.1 s bins; *n* = 15 mice. (E) Mean RTs increased with P_beep_. Data are represented as mean ± SEM across mice; *n* = 15 mice. Statistical significance was assessed using a linear mixed-effects model with mouse as a random intercept; *p* < 10^−6^. (F) First-lick probability distributions aligned on beep onset, shown for 200 ms (light), 400 ms (medium), and 600 ms (dark) beeps. Data are represented as mean ± SEM across mice; 0.1 s bins; *n* = 15 mice. (G) P_response_, defined as the probability of responding within 1 s of beep onset, decreased with Pbeep, increased with beep duration, and showed a significant Pbeep × duration interaction. Data are represented as mean ± SEM across mice; *n* = 15 mice. Statistical significance was assessed using a linear mixed-effects model with mouse as a random intercept; P_beep_: *p* < 10^−6^, duration: *p* < 10^−6^, interaction: *p* = 1.01 × 10^−4^.

We analyzed data from 15 mice that showed stimulus-dependent responding (see Methods, Figure S1). To quantify how long mice waited before responding, we defined an RT measure anchored to the onset of the sustained beep that continues into the “Go” window (see Methods). Trials in which a lick occurred after this sustained-beep onset, whether rewarded or premature, were assigned an RT, whereas trials in which the mouse licked before this onset were excluded from RT analyses (Figure 2A).

To verify that mice were responding to sound rather than chance timing, we compared the mean RT distribution to a shuffled baseline that preserves lick times but randomizes the underlying beep streams (see Methods). The mean distribution of RTs exceeded the shuffled distribution at short latencies and fell below it at longer latencies (Figure 2B, mean ± SEM across mice), indicating sound-locked responding. Consistent with this, mice performed above a shuffled baseline and showed a modest dependence of performance on P_beep_ (Figure S1).

We next asked whether cue reliability (P_beep_) systematically shifts responses. An example session illustrates how response timing was modulated by P_beep_: RTs cluster at shorter latencies in the lower-P_beep_ condition and extend to longer latencies in the higher-P_beep_ condition (Figure 2C). RT distributions across all mice showed a clear rightward shift as P_beep_ increased (Figure 2D), indicating mice waited longer before responding in higher P_beep_ blocks. A linear mixed-effects model confirmed that mean RTs increased significantly with P_beep_ across animals (Figure 2E; *p* = 1.55 × 10^−13^).

Having established sound-locked licking and P_beep_-dependent waiting to continuous beeps, we next asked how responses were shaped by silences, preceded by varying beep lengths. We analyzed responses to short beeps that occurred during the premature timeout and the ITI. These are segments in which licking does not alter the sound stream, giving us access to uninterrupted patterns of stimulus. Within and across P_beep_ contexts, we identified isolated 200, 400, and 600 ms beeps and computed lick probability aligned to beep onset. Response distributions revealed that longer beeps elicited stronger and more sustained increases in lick probability, whereas intervening silences produced clear decreases (Figure 2F). This decrease is most evident at intermediate P_beep_ values (≈0.2–0.6), where lick probability rises during the beep and then systematically drops following beep offset, indicating that licking is reduced during the subsequent silent period rather than at a fixed time from beep onset.

To quantify these effects, we defined P_response_ as the probability of licking in the first second after beep onset (Figure 2G) and fit a linear mixed-effects model with fixed effects of P_beep_, duration, and their interaction. P_beep_ had a strong negative effect (*p* = 1.95 × 10^−38^); lick probability increased with beep duration (*p* = 1.50 × 10^−11^), and the duration effect was stronger at lower P_beep_ (*p* = 1.01 × 10^−4^).

Together, these analyses show that mice wait longer as P_beep_ increases, demonstrating a robust contextual influence of cue reliability on behavior. Lick probability scales with beep duration and is suppressed by silence, consistent with a ramp-and-reset DV.

### Sensory evidence drives rapid waiting-time adaptation

**Figure 3:**
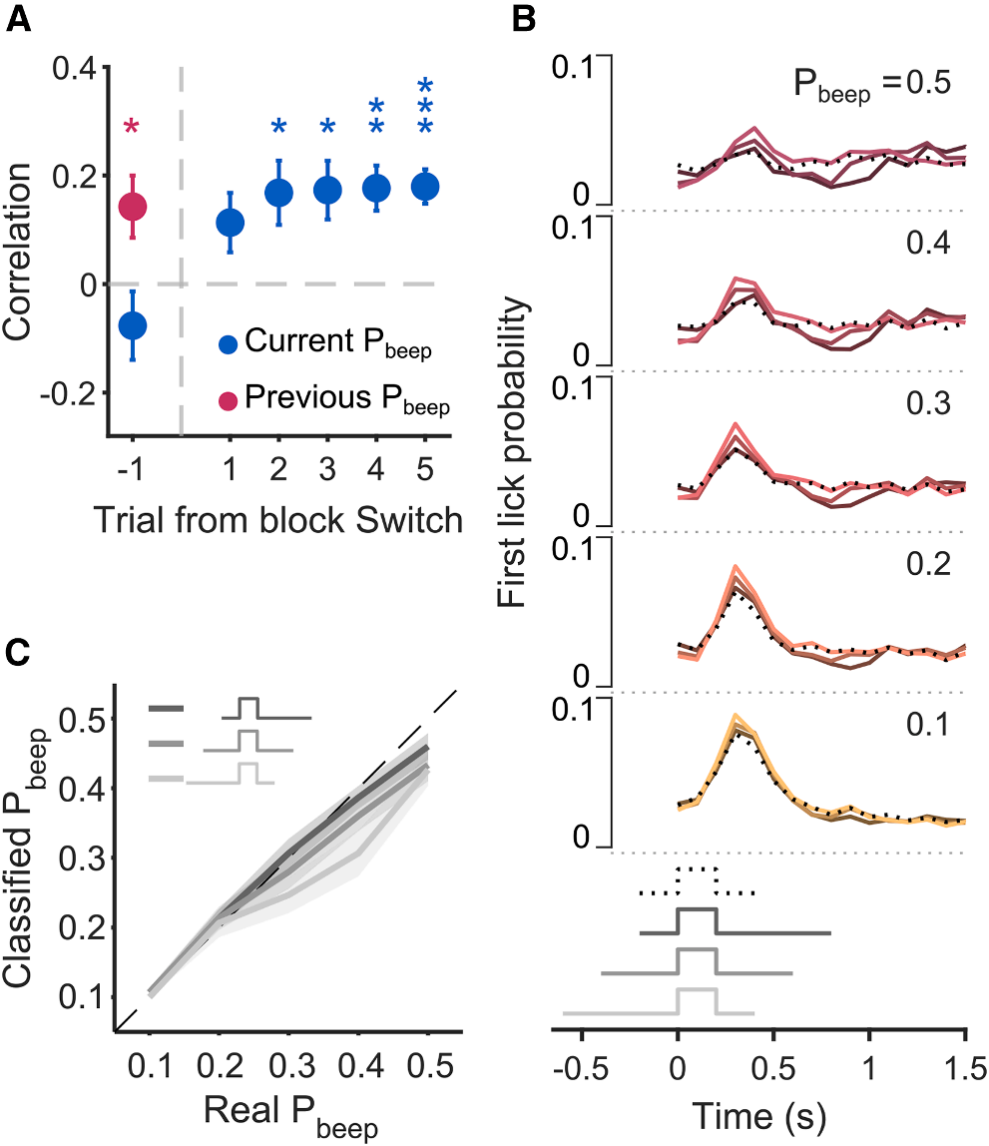
Mice adapt their waiting behavior according to sensory statistics (A) Correlation between response times and P_beep_ on trials surrounding a block switch. Correlations were computed within each mouse and tested against zero across animals using two-tailed one-sample t-tests; *p* values for current P_beep_ correlations were FDR-corrected across plotted trial offsets. Points show mean ± SEM across mice; *n* = 15 mice. Blue points show correlations with the current-block P_beep_, and the red point shows the correlation with the previous-block P_beep_ on the last trial before the switch. ∗*p* < 0.05, ∗∗*p* < 0.01, and ∗∗∗*p* < 0.001. (B) First-lick probability distributions for 200 ms beeps preceded by 200, 400, or 600 ms of silence (dark to light). Reference distributions for 200 ms beeps are shown as black dotted lines. Data are represented as mean ± SEM across mice; 0.1 s bins; *n* = 15 mice. (C) Kullback–Leibler (KL) divergence-based classification of these distributions relative to the 200 ms reference distributions. Classified P_beep_ decreased with increasing preceding silence. Data are represented as mean ± SEM across mice; *n* = 15 mice. Statistical significance was assessed using a linear mixed-effects model with preceding-silence pattern, P_beep_, and their interaction as fixed effects and mouse as a random intercept. Classified P_beep_ decreased with increasing preceding silence (*p* = 0.0027), increased with real P_beep_ (*p* < 10^−6^), and showed no significant interaction between preceding silence and P_beep_ (*p* = 0.146).

A central question is whether the observed timing adaptation reflects rapid adjustment based on the sensory stream or slow adjustment driven by reward feedback. If mice primarily relied on feedback, response timing should update gradually within a block. By contrast, adaptation based on sensory statistics predicts a rapid shift immediately after a block switch. We therefore examined how quickly RTs realigned to the new context. Correlations between P_beep_ and RTs increased immediately after block switches, reaching significance by the second trial (Figure 3A). This suggests that mice begin adjusting their waiting strategy based on sensory statistics, but leaves open whether the adaptation observed on the second trial reflects feedback to the lick on the first trial, or instead arises from continued exposure to the stimulus stream.

We again leveraged beeps occurring during the premature timeout and ITI segments to test whether stimulus features themselves drove adaptation. Specifically, we extracted first-lick distributions for three matched patterns of stimulus that each contained a 200 ms beep but differed in the silence preceding (200, 400, or 600 ms) and the silence following the beep (600, 400, or 200 ms, respectively). This construction isolates the effect of preceding silence on the response to a beep, while balancing total response opportunity across stimulus patterns.

Under a ramping/resetting DV, silence should reset activity away from threshold, yet in this task longer preceding silence also signals lower P_beep_. Within blocks of matched P_beep_ (for P_beep_ = 0.1–0.5 where these patterns occurred frequently), longer preceding silences elicited stronger responses to the subsequent beep (Figure 3B). This within-context effect mirrored the across-context relationship, such that responses following longer silences more closely resembled those observed at lower P_beep_ values.

To quantify this, we compared these pattern distributions to reference distributions obtained in Figure 2F (the 200 ms beep). For each pattern distribution, we computed the Kullback–Leibler (KL) divergence to every reference distribution and classified it to the P_beep_ of the reference distribution with the minimal divergence. We found that the classified P_beep_ value decreased as the preceding silence increased (Figure 3C). A mixed-effects model confirmed that classified P_beep_ decreased with longer preceding silence (*p* = 0.0027). P_beep_ showed a positive main effect (*p* < 10^−6^); the interaction was not significant (*p* = 0.146). Because silences in the sensory stream were associated with responses that more closely resembled low-P_beep_ behavior, these results indicate that mice use ongoing sensory input, rather than only reward feedback, to guide context-dependent adjustments in behavior.

### Context adaptation is captured by drift rate modulation in an evidence accumulation model

**Figure 4:**
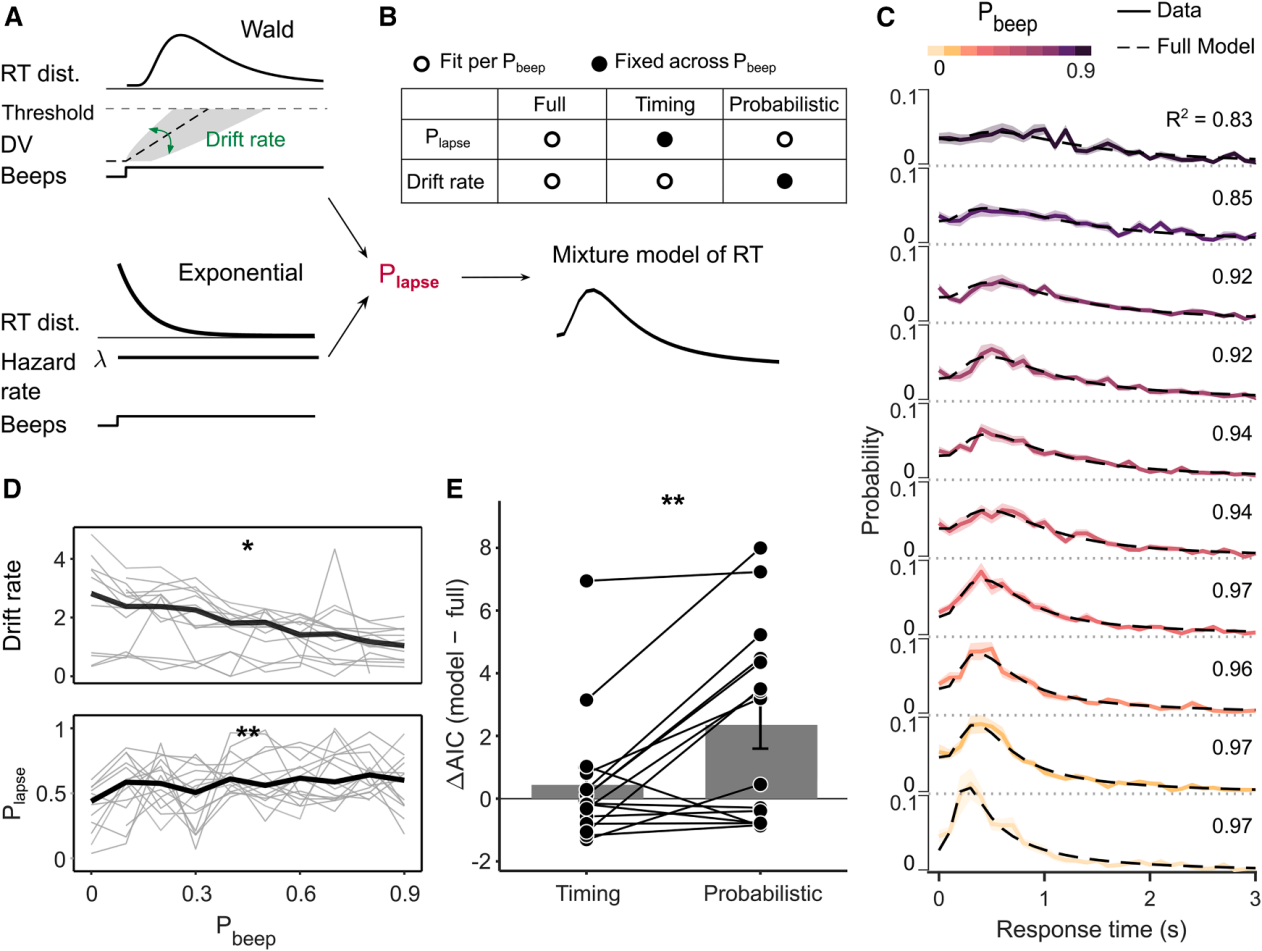
Context adaptation is captured by drift-rate modulation in an evidence-accumulation model (A) Schematic of the response-time mixture model combining a Wald distribution for accumulation-driven responses and an exponential distribution for stimulus-independent licks. (B) Structure of the full model and the restricted variants in which timing parameters (drift rate) or probabilistic parameters (lapse probability) were allowed to vary across P_beep_. (C) Model fits to response-time distributions across P_beep_ conditions. Data are represented as mean ± SEM across mice; 0.1 s bins; *n* = 15 mice. R^2^ values indicate the fraction of variance explained in each condition. (D) Parameter estimates across P_beep_. Thin gray lines show individual mice; thick black lines show population median. Drift rate decreased with P_beep_ and lapse probability increased with P_beep_, assessed using linear mixed-effects models with mouse as a random intercept; *n* = 15 mice; ∗*p* = 0.0156 and ∗∗*p* = 0.0040. (E) Model comparison using Akaike Information Criterion (ΔAIC relative to the full model; lower values indicate better fits). The timing model provided a significantly better fit than the probabilistic model, assessed using a paired two-tailed *t* test across mice; bars show mean ± SEM, *n* = 15 mice; ∗∗*p* = 0.0064.

To formalize how context (P_beep_) shapes behavior and to probe the underlying mechanism of context adaptation, we modeled RTs as arising from an evidence-accumulation process with occasional lapses (Figure 4A). Examining the behavior, we found that mice produced stable response latencies within blocks, yet also emitted occasional stimulus-independent licks. To separate accumulation-driven timing from these lapses and to identify the mechanism underlying context adaptation, we modeled the RT distributions using a two-component mixture: a Wald distribution describing one-sided accumulation to a bound,^1^ and an exponential distribution capturing random, lapse-like responses. Lapses are a well-documented feature of rodent decision making,^16^^,^^17^^,^^18^^,^^19^ and so this mixture model provides a natural account of both instructed and uninstructed responding.

While the mixture model contains four parameters (see Methods, Figure S2), here we focus on two that capture distinct behavioral mechanisms: the drift rate, which controls the speed of evidence accumulation, and the lapse probability (P_lapse_), which determines the weight of random versus accumulation-driven responses.

We considered two candidate mechanisms of context adaptation, termed “Timing” and “Probabilistic.” In the “Timing” adaptation, mice shift *when* they respond by modulating the speed of evidence accumulation as a function of P_beep_. In our model, two of the accumulation parameters, the drift rate and decision threshold, both influence response timing and can partially compensate for each other (Figure S3). To avoid redundancy, we compared models in which each was fixed or allowed to vary across contexts. This analysis confirmed that the two parameters are interdependent, but that drift variation provided a better account of the data. To isolate the speed of accumulation, we therefore fixed the threshold and focused on context-dependent changes in drift.

In the “Probabilistic” model, mice adapt *how often* they engage in the accumulation process versus stimulus-independent licks. This is implemented as a context-dependent change in P_lapse_ while keeping accumulation fixed. This form of adaptation is motivated by the idea that if animals cannot modulate their response timing, a fixed latency that is adequate in low-P_beep_ contexts becomes maladaptive in high-P_beep_ contexts, leading to unrewarded responses. Such contingency degradation would reduce the effective utility of accumulation, leading animals to rely on it less frequently, manifesting as an increase in lapse probability.

These mechanisms make different qualitative predictions for the RT distribution: changes in timing (drift) shift the accumulation-driven peak and its spread, whereas Probabilistic changes (lapse) primarily redistribute probability between the accumulation-driven peak and the rest of the distribution (Figure S4A). These qualitative signatures provide a basis for interpreting how model parameters relate to the observed data.

We therefore compared restricted models in which one of these components varies with context while the other is held fixed (Figure 4B), and then asked which account better captured the observed distributions relative to the full model in which both parameters are fit for each P_beep_ separately.

The full model captured the RT distributions across P_beep_ conditions with high accuracy (Figure 4C). We next asked which parameter accounted for the observed context adaptation. At the group level, both parameters covaried with P_beep_ context (linear mixed-effects models; drift *p* = 0.0156, lapse *p* = 0.004; Figure 4D). Inspection across animals revealed clear heterogeneity: Most mice (11 of 15) showed context-dependent changes primarily in the drift rate with little change in lapse probability, whereas a minority (4 of 15) showed the opposite pattern (Figure S5). To adjudicate mechanisms, we compared restricted models that allowed only the accumulation component or only the lapse component to vary with P_beep_. Akaike Information Criterion (AIC) favored the accumulation-varying (timing) model over the lapse-varying (probabilistic) model (paired *t* test on ΔAIC relative to the full model, *p* = 0.0064; Figure 4E). Comparison of the full model parameters, including the exponential component of the lapse process (Figure S6) showed that variation in drift rate accounted for the largest improvement in model fit, confirming that context adaptation is primarily driven by changes in the speed of evidence accumulation. These results were robust to alternative censoring procedures (Figure S7).

### Orbitofrontal cortex contributes to context-dependent timing under ambiguity

**Figure 5:**
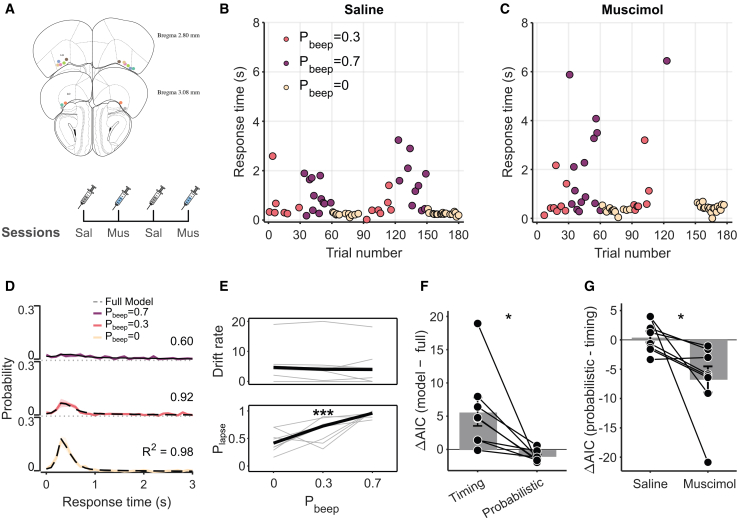
Orbitofrontal cortex contributes to context-dependent timing under ambiguity (A) Schematic of bilateral muscimol injections targeting orbitofrontal cortex (OFC; top) and the injection schedule (bottom). Mice received alternating saline and muscimol sessions; *n* = 8 mice. (B) Response times across P_beep_ conditions in an example saline session. (C) Response times across P_beep_ conditions in an example muscimol session from the same mouse. (D) Fits of the full mixture model to response-time distributions in the muscimol condition. Data are represented as mean ± SEM across mice; 0.1 s bins; *n* = 8 mice. Under OFC inactivation, distribution peaks did not shift with P_beep_, but their amplitude decreased. (E) Parameter estimates across P_beep_ under OFC inactivation. Thin gray lines show individual mice; thick black lines show the population median. Drift rate no longer varied significantly with P_beep_, whereas lapse probability increased with P_beep_, assessed using linear mixed-effects models with mouse as a random intercept; *n* = 8 mice; drift rate: *p* = 0.107, lapse probability: *p* = 2.95 × 10^−6^. (F) Model comparison using Akaike Information Criterion (ΔAIC relative to the full model; lower values indicate better fits). Under OFC inactivation, the lapse-modulation model provided a better fit than the drift-modulation model, assessed using a paired two-tailed *t* test across mice; bars show mean ± SEM, *n* = 8 mice; ∗*p* = 0.019. (G) The relative advantage of the lapse-modulation model over the drift-modulation model (ΔAIC_prob_-ΔAIC_tim_) was larger in muscimol than in saline sessions, assessed using a paired two-tailed *t* test across mice; bars show mean ± SEM, *n* = 8 mice; ∗*p* = 0.038.

The OFC has been linked to representing latent task states^14^^,^^20^ and guiding behavior when external cues are ambiguous,^12^ but whether it supports flexible tuning of evidence accumulation remains unclear. We tested this by bilaterally inactivating lateral OFC with muscimol (a GABA-A agonist; Figure 5A). We analyzed data from eight mice that completed alternating saline and muscimol sessions (Figure S1).

During saline, mice continued to adjust response timing with context, waiting longer in blocks with higher P_beep_, as illustrated in an example session (Figure 5B). OFC inactivation disrupted this pattern: Mice exhibited either near-fixed latency responses or irregular licking patterns. In a representative session, the animal alternated between fixed-latency licks and scattered responses, and eventually ceased licking altogether for more than one block before resuming vigorous licking at P_beep_ = 0, where the cue is unambiguous (Figure 5C). A similar P_beep_ -dependent reduction in licking was observed across animals and was driven by reward feedback (Figure S8). Critically, however, when mice did respond, they no longer adjusted their response timing. Consistent with this, the mean RT distributions no longer showed a systematic peak shift with P_beep_; instead, the peak remained at a similar latency and its amplitude scaled with context, a signature more consistent with increased lapse-like responding (Figures 5D, S4, and S9). We quantified these changes with the same Wald-exponential mixture model used above. Under muscimol, drift rate no longer showed significant modulation with P_beep_ (linear mixed-effects model, *p* = 0.1069), whereas lapse probability increased with P_beep_ (*p* = 2.95 × 10^−6^; Figure 5E). Model comparison confirmed a change in the underlying strategy: Across mice, the probabilistic model fit better than the timing model during muscimol (ΔAIC, paired *t* test, *p* = 0.0193; Figure 5F). Under muscimol, the AIC difference between the probabilistic and timing models (ΔAIC = AIC _probabilistic_ −AIC_timing_) was significantly more negative than in saline, meaning the data provided stronger evidence for the probabilistic model during inactivation (paired *t* test, *p* = 0.0378; Figure 5G). To examine this change at the parameter level, we compared all model parameters in the muscimol dataset (Figure S6). This analysis revealed that variation in lapse probability (P_lapse_) provided the largest improvement in model fit, outperforming both the drift and exponential parameters. These results confirm that, during OFC inactivation, context adaptation is better captured by changes in lapse probability rather than in accumulation dynamics. Together, this suggests that OFC contributes to maintaining structured context-dependent timing under ambiguity.

### Removing the benefit of prolonged waiting increases lapse-like responding

**Figure 6:**
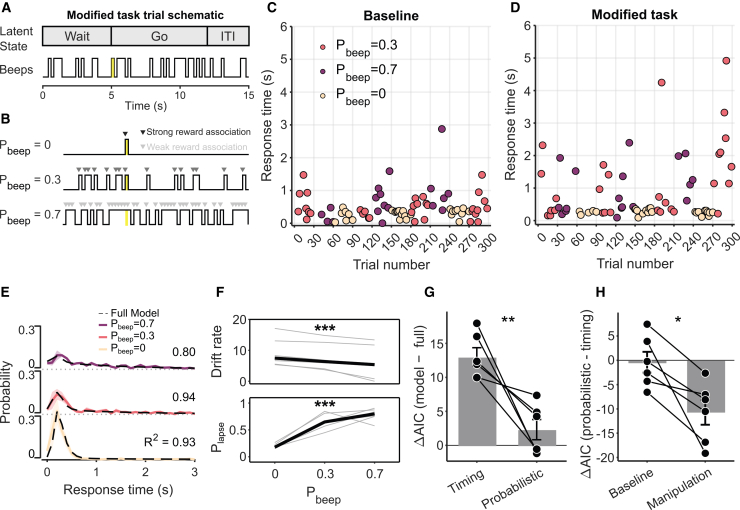
In a control task without prolonged waiting demands, behavior is better captured by lapse-modulation than drift-modulation (A) Schematic of the modified task. The “Go” state began with a 200 ms beep followed by a stochastic sequence of beeps drawn according to P_beep_. (B) In this task variant, P_beep_ still modulated cue reliability: in low-P_beep_ blocks, beeps strongly predicted reward, whereas in high-P_beep_ blocks, each beep was less informative. (C) Response times across P_beep_ conditions in an example session of the baseline task. (D) Response times across P_beep_ conditions in an example session of the modified task for the same mouse. (E) Fits of the full mixture model to response-time distributions across P_beep_ in the modified task. Data are represented as mean ± SEM across mice; 0.1 s bins; *n* = 6 mice. Distribution peaks remained aligned across contexts, while amplitudes decreased with increasing P_beep_. (F) Parameter estimates across P_beep_ in the modified task. Thin gray lines show individual mice; thick black lines show the population median. Drift rate decreased with P_beep_ and lapse probability increased with P_beep_, assessed using linear mixed-effects models with mouse as a random intercept; *n* = 6 mice; drift rate: *p* = 5.82 × 10^−6^, lapse probability: *p* = 3.74 × 10^−6^. (G) Model comparison using Akaike Information Criterion (ΔAIC relative to the full model; lower values indicate better fits). In the modified task, the lapse-modulation model provided a better fit than the drift-modulation model, assessed using a paired two-tailed *t* test across mice; bars show mean ± SEM, *n* = 6 mice; ∗∗*p* = 0.0084. (H) The relative advantage of the lapse-modulation model over the drift-modulation model was larger in the modified task than in the baseline task, assessed using a paired two-tailed *t* test across mice; bars show mean ± SEM, *n* = 6 mice; ∗*p* = 0.0137. ∗*p* < 0.05, ∗∗*p* < 0.01, and ∗∗∗*p* < 0.001.

We hypothesized that OFC inactivation disrupted the structured context-dependent adjustment of response timing. To test this, we designed a modified version of the task in which adjusting waiting time across P_beep_ no longer conferred a benefit, predicting that behavior in this version would mimic that observed during OFC inactivation. In the modified version, the “Go” state always began with a 200 ms beep, after which the 7 s response window contained stochastic beeps drawn according to P_beep_ (Figure 6A). Thus, only the onset of the tone carried information about reward availability; waiting for a longer beep provided no additional advantage. This manipulation preserved the usual relationship between P_beep_ and cue reliability as sounds are more informative at lower P_beep_, but removed the incentive to prolong waiting (Figure 6B). To examine behavior under this modified task, we trained a new batch of mice. These animals were first trained on the standard version of the task using the same protocol as the muscimol cohort, and were then tested for two days on the modified version. We analyzed data from 6 mice in the modified task (Figure S1). In an example baseline session, there is context-dependent waiting (Figure 6C). In the modified task, the same animal produced fixed-latency or scattered licking patterns (Figure 6D). Consistent with lapse rate context adaptation, the peaks of the RT distributions aligned across P_beep_ and primarily changed in amplitude rather than shifting in time (Figure 6E).

We fit the same Wald-exponential mixture model. The model continued to capture RT distributions well. Parameter estimates indicated a strategy shift (Figure 6F): Both drift-rate and lapse components varied with context (linear mixed-effects models; drift *p* = 5.8 × 10^−6^; lapse *p* = 3.7 × 10^−6^). The residual dependence of drift rate on P_beep_ likely reflects carryover from prior training on the original task where mice learned to modulate accumulation, rather than an advantage in the modified task, in which extended waiting provides no benefit. Model comparison confirmed that the probabilistic model better explained behavior in the modified task (ΔAIC relative to the full model, paired *t* test, *p* = 0.0084; Figure 6G). Moreover, the AIC difference between probabilistic and timing models (ΔAIC = AIC _probabilistic_ −AIC_timing_) was more negative in the modified task than in baseline, meaning stronger evidence for the probabilistic account under the manipulation (paired *t* test, *p* = 0.0137; Figure 6H). To assess this shift more comprehensively, we compared all model parameters in the modified-task dataset (Figure S6). This analysis again identified P_lapse_ as the parameter contributing most strongly to model fit, significantly outperforming both drift and exponential components. These results confirm that, when the task removes the benefit of longer waiting, context adaptation is best captured by changes in lapse probability rather than in accumulation dynamics.

Together, these results show that accumulation adjustment is deployed when longer waiting can improve performance. When the task removes that benefit, mice shift to a different strategy that modulates lapse probability, paralleling the pattern observed with OFC inactivation.

## Discussion

Our results reveal that mice flexibly adapt their response timing to the reliability of sensory cues, and that the OFC contributes to maintaining this structured, context-dependent control of behavior. By decoupling the physical structure of auditory inputs from their informativeness, we show that behavioral adjustments arise from changes in decision policy rather than from the immediate sensory features of the stimulus stream. This establishes a new behavioral paradigm for studying how animals adjust timed decisions when the informativeness of sensory evidence changes across contexts.

In our task, mice adapt their RTs to cue reliability, waiting longer when beeps are more ambiguous. Some studies examining latent-state inference or flexible behavior have used reward outcomes as the information to be integrated.^21^^,^^22^ Such paradigms may confound policy adaptation with well-established effects of reward on movement vigor and response latency.^23^^,^^24^^,^^25^ Here, adaptation was driven by the sensory stream itself. Mice adjusted quickly following block switches, indicating that behavior was shaped rapidly by ongoing sensory statistics rather than solely by slow, feedback-driven learning. Analysis of specific stimulus patterns further supports this interpretation. Under a simple ramp-and-reset account, silence should reset the DV by pushing it away from threshold, yet we found that beeps following longer silences elicited faster responses. This effect is consistent with longer silences signaling lower P_beep_, and suggests that animals use ongoing sensory statistics to guide context-dependent behavioral adjustments.

RTs have long served as a window into hidden cognitive operations.^26^^,^^27^^,^^28^ Building on this tradition, contemporary approaches model full RT distributions with process-linked forms^29^^,^^30^^,^^31^ designed to adjudicate among mechanistic hypotheses. Here, we leverage this approach to probe the basis of behavioral adaptation in our task. Because the model captures the empirical RT distributions well, parameterized fits allow us to ask which model component best accounts for adaptation (e.g., changes in accumulation rate versus lapse propensity). In this framework, the parameter that most strongly captured the context-dependent shifts in response timing was the drift rate. Our modeling results provide a computational account of how mice adapt their decision-making to cue reliability.

A consideration in interpreting these results is that parameters of evidence accumulation models, such as drift rate and decision threshold, can partially trade off in shaping RT distributions. In our analysis, we fixed the threshold to isolate changes in accumulation dynamics and found that allowing drift rate to vary provided a better account of the data than allowing threshold variation alone. However, this modeling choice constrains how adaptation is expressed, and alternative parameterizations could, in principle, capture similar behavioral effects. Accordingly, our results are best interpreted as reflecting changes in accumulation dynamics, rather than uniquely identifying drift rate as the sole underlying mechanism.

Related work on evidence accumulation in non-stationary environments has modeled adaptive behavior using mechanisms such as leaky integration, in which older evidence is progressively discounted over time.^32^^,^^33^^,^^34^ While such mechanisms can produce adaptive changes in accumulation dynamics, our results suggest that behavior in this task cannot be explained solely by local accumulation and decay processes. In particular, prolonged silent intervals facilitated subsequent responses rather than simply weakening accumulated evidence (Figure 3), making the observed adaptation difficult to reconcile with simple leaky integration dynamics. Consistent with these observations, our model captures adaptation in this task as context-dependent modulation of accumulation rate.

Inactivating the OFC reduced this form of timing adaptation and revealed a different pattern of behavior that, in the model, was captured primarily as an increase in lapse probability. We interpret the increase in P_lapse_ as reflecting a shift in the balance between accumulation-based responding and an alternative, less stimulus-dependent mode of behavior. Notably, OFC inactivation also produced broader behavioral effects, including reduced overall responding, increased outcome sensitivity, and greater variability within blocks, indicating a more general disruption of structured behavior. A central question is how to interpret the effect of OFC inactivation in light of competing theories of OFC function. Several frameworks emphasize distinct roles for OFC, including representing latent task states or “cognitive maps” under partial observability,^12^^,^^35^ encoding value to guide choice,^36^^,^^37^^,^^38^^,^^39^^,^^40^ supporting model-based and goal-directed behavior,^22^^,^^41^^,^^42^^,^^43^ and contributing to timing and waiting.^44^^,^^45^^,^^46^^,^^47^

In our task, OFC inactivation did not eliminate context sensitivity altogether, as mice still behaved differently across P_beep_ blocks. Instead, the disruption reduced structured context-dependent control of behavior. This pattern is consistent with OFC contributing to inference over latent task structure, although it does not distinguish between a failure to represent context, a failure to use context to guide behavior, or a failure to maintain a stable context-dependent policy.

This inference-based interpretation is further supported by how the effects scaled with cue ambiguity. P_beep_ determines how ambiguous individual cues are, and under OFC inactivation, mice performed best in the P_beep_ = 0 condition, where cues are unambiguous, but exhibited markedly elevated lapse rates as ambiguity increased. Such a profile is in line with OFC being necessary for inferring whether a sustained beep reflects a true state transition or a spurious sequence of beeps. At the same time, alternative interpretations remain possible. For example, OFC inactivation may impair the maintenance of block context, leading animals to adopt a more uniform or unstable response policy across conditions. In this case, the observed changes in response timing and lapse behavior could arise secondarily from a failure to maintain structured, context-dependent control, rather than from a direct impairment in tuning accumulation parameters.

Recent work provides a potential circuit-level substrate for this form of context-dependent adaptation. Projections from OFC to auditory cortex have been shown to carry task-related information and modulate sensory representations according to behavioral demands.^48^ Similarly, OFC inputs to somatosensory cortex support flexible use of sensory cues during reversal learning.^39^ These findings suggest that OFC may influence evidence accumulation by modulating the impact of incoming sensory signals at early stages of processing. In our task, such top-down signals could adjust how strongly individual beeps drive accumulation depending on estimated cue reliability, providing a potential circuit mechanism for context-dependent modulation of sensory evidence.

To further interpret the effect of OFC inactivation, we designed a control task in which adapting waiting times offered no advantage. As predicted, animals performing this modified task behaved similarly to OFC-inactivated mice in the original task, showing little adjustment of their RTs across contexts. This parallel suggests that the behavioral profile observed during OFC inactivation resembles the pattern seen when prolonged waiting is no longer beneficial.

Together, our findings establish a behavioral framework for studying how animals adapt timed decisions when the reliability of sensory evidence changes across contexts. In this setting, mice adapt their decision policy, lengthening waiting times under ambiguity, and our model captures this flexibility as adjustments in accumulation dynamics. OFC emerged as a key circuit supporting structured, context-dependent control of behavior in this task. OFC inactivation reduced this structured timing adjustment and was captured in the model by increased lapse-like responding, highlighting the contribution of OFC to using task structure to guide behavior.

### Limitations of the study

A limitation of this study is that the RT model provides a computational account of the observed behavioral distributions, but does not uniquely identify the underlying decision mechanism. In particular, the model captures context-dependent timing as modulation of an accumulation/timing parameter, yet alternative parameterizations or related models could potentially account for similar behavioral effects. Thus, the model should be interpreted as a framework for describing and comparing behavioral patterns across conditions, rather than as definitive evidence for a specific neural implementation of evidence accumulation.

A second limitation is that muscimol inactivation does not resolve the circuit mechanism through which OFC contributes to behavior. Although injections were targeted to OFC, local spread to nearby tissue is possible, and the manipulation does not identify the relevant cell types, projections, or temporal epochs. The inactivation results therefore support a contribution of OFC to structured context-dependent timing under ambiguity, but do not specify the precise neural pathway.

Finally, the behavioral effects do not distinguish whether OFC contributes by representing the current context, maintaining a stable context-dependent policy, or using contextual information to guide action. Future work combining neural recordings with temporally and circuit-specific manipulations will be needed to distinguish these possibilities.

## Resource availability

### Lead contact

Requests for further information and resources should be directed to and will be fulfilled by the lead contact, Dr. Eran Lottem (eran.lottem@mail.huji.ac.il).

### Materials availability

This study did not generate new unique reagents.

### Data and code availability

• All data reported in this paper will be shared by the lead contact upon request.
• This paper does not report original code.
• Any additional information required to reanalyze the data reported in this paper is available from the lead contact upon request.

## Acknowledgments

This work was supported by a grant from the 10.13039/501100003977Israel Science Foundation (grant no. 1269/20 [to E.L.]), the 10.13039/501100000324Gatsby Charitable Foundation (to J.K.), and the National Institute of Psychobiology (to J.K.). E.L. is the incumbent of the Sachs Family Faculty Development Chair in Brain Sciences.

We thank Mati Joshua and Raunak Basu for comments on the manuscript.

## Author contributions

E.L. conceived the study. E.L., H.R., R.R., J.S., and G.M. developed the methodology. E.L., G.M., J.S., and H.R. developed hardware and software tools. E.L. and G.M. performed the formal analyses. J.S. and J.K. computed the analytical reward rate. H.R. and G.M. performed the experiments. R.R. provided resources and coordinated project administration. G.M. wrote the original draft. All authors reviewed and edited the manuscript. E.L. and J.K. supervised the project and acquired funding.

## Declaration of interests

The authors declare no competing interests.

## Declaration of generative AI and AI-assisted technologies in the writing process

During the preparation of this work, the authors used OpenAI’s ChatGPT to assist with text editing and revision, as well as to generate and refine visualization and statistical analysis code. All AI-generated content was reviewed, verified, and edited by the authors, who take full responsibility for the final manuscript and all analyses.

## STAR★Methods

### Key resources table

**Table:** 

REAGENT or RESOURCE	SOURCE	IDENTIFIER
**Chemicals, peptides, and recombinant proteins**		

Muscimol	Cayman Chemical	CAS: 2763-96-4; Cat# 13667

**Experimental models: Organisms/strains**		

C57BL/6J male mice	The Jackson Laboratory	Strain #: 000664; RRID: IMSR_JAX:000664

**Software and algorithms**		

MATLAB R2023b (data analysis)	MathWorks	https://www.mathworks.com/products/matlab.html
Cupid MATLAB toolbox (Modeling)	Jeff Miller, GitHub	https://github.com/milleratotago/Cupid
Bpod behavior system, version 1.8.1	Sanworks	RRID: SCR_015943
Bonsai, version 2.6.3	Bonsai Foundation	RRID: SCR_021512

### Experimental model and study participant details

#### Animals

All procedures were approved and performed in accordance with the Institutional Animal Care and Use Committee (IACUC) of The Hebrew University of Jerusalem under protocol number NS-23-17260-4. Experiments were conducted using C57BL/6J male mice (age: 2–4 months; weight: 20–30 g) under a water restriction protocol. Only male mice were included in the study; therefore, sex was not evaluated as a biological variable. Body weight was monitored daily, and supplemental water was provided as needed to ensure that weight did not fall below 80% of the initial weight. Mice had *ad libitum* access to food in their home cages and they were housed under a 12-h light/dark cycle. Training, as well as testing, occurred during the light period.

### Method details

#### Head-bar surgery

To implant head bars for head fixation, mice were anesthetized using 2–3% isoflurane (SomnoSuite, Kent Scientific, USA) and placed in a stereotaxic apparatus (KOPF 962, David Kopf Instruments, USA). Meloxicam (2 mg/kg) was injected subcutaneously for systemic analgesia and 0.1 mL lidocaine from a 2% solution was injected subcutaneously before incising the scalp and exposing the skull. For mice assigned to orbitofrontal cortex (OFC) manipulation experiments, the OFC was marked prior to head-bar implantation using stereotaxic coordinates (AP: +2.9 mm; ML: ±1.25 mm) relative to bregma to guide future craniotomy. A 20 mm long and 3 mm wide head-post was then cemented to the skull horizontally using dental cement (C&B Super-bond, Sun Medical, Japan) and secured using dental resin (Pi-ku-plast, Bredent, Germany). Post-operative analgesia was administered and mice were allowed to recover for 2 to 3 days before beginning a 5-day water-restriction schedule in preparation for training.

#### Muscimol infusions

Mice were anesthetized with 2–3% isoflurane, and a bilateral craniotomy (∼1 mm in diameter) was made over the OFC one day before the first manipulation session. Then the craniotomy site was covered with a silicone seal (KWIK-CAST, WPI, USA) to prevent drying and infection. On each of the subsequent four days, mice were anesthetized and injected using a Hamilton syringe with either saline (230–250 nL) or muscimol (a GABA-A agonist; Cayman Chemicals, USA) (230–250 nL of 1 mg/mL solution in normal saline at a rate of 100 nL/min) targeting a depth of 1.8 mm from the dura. To enable post hoc verification of injection sites, muscimol was mixed with Chicago sky blue dye (25 mg/mL final concentration in saline) and the needles were coated with lipophilic fluorescent dyes (DiI or DiO; Thermo Fisher Scientific). After each injection, animals were returned to their home cage and allowed to recover for 10–15 min and to permit drug diffusion before behavioral testing began.

#### Histology

For histological analysis, mice were deeply anesthetized using isoflurane followed by I.P. injection of Pentobarbital (200 mg/kg) and perfused transcardially with 4% formalin solution in 1×PBS. After removing the brains, they were left for 24 h in 4% formalin solution in PBS, then transferred to 1×PBS. Brains were sliced in 100 μm coronal sections using a vibratome (Leica VT 1000 S), collected on a slide and mounted with Vectashield (Vector Laboratories). Fluorescence images were acquired with a microscope (Olympus IX83) using a 4× objective. Injection sites were estimated by identifying the dye traces and comparing the shape of the brain sections with the Allen Mouse Brain Atlas.

#### Change detection task

Head-fixed mice were trained to perform an auditory change detection task. In each trial, mice were exposed to a train of auditory beeps presented in one of two hidden states: A ‘Wait’ state and a ‘Go’ state. During the ‘Wait’ state, each 0.2 s time bin contained either a beep that occurred with a fixed probability (P_beep_) or a silence with probability (1- P_beep_). After a delay drawn from an exponential distribution (0.8 + 0.2⌊*x*⌋ seconds, where x ∼ exp (λ = 0.1)), the state transitioned to the ‘Go’ state, where beeps occurred continuously (i.e., with probability = 1 in each 0.2 s bin). Licking during the ‘Wait’ state was considered premature and triggered a 7 s timeout period (Premature state) before moving to the inter-trial interval (ITI state). In contrast, licking in the ‘Go’ state triggered a reward (4–6 μL of water) and caused an immediate transition to the ITI. Cue reliability was manipulated across trial blocks (30–50 trials each) by varying P_beep_, the probability of beeps in the ‘Wait’ state. Low P_beep_ values resulted in high certainty (sparser misleading beeps), allowing mice to identify state transitions more easily. High P_beep_ values increased uncertainty, requiring more evidence (i.e., longer beep sequences) to correctly infer the ‘Go’ state.

Auditory stimuli were harmonic chords consisting of pure tones at 2, 4, 6, 8, and 16 kHz, presented at 70 dB SPL through a speaker adjacent to the animal. The task was controlled using the Bpod behavior system (Sanworks, USA). Licking behavior was monitored using a 226-fps camera (Blackfly BFS-U3-16S2M-CS, FLIR, USA) with a Tamron M118FM1-6 lens (Tamron, Japan) and detected online using Bonsai software.

#### Behavioral cohorts and training progression

Seventeen mice were initially trained at a single P_beep_ (0.2 or 0.3) to establish the basic state-transition rule. The training then advanced to sessions containing multiple P_beep_ blocks with values ranging from 0 to 0.9 in 30–50 trials per block. A total of fifteen animals responded to sound in the task and were included in the study (Figure S1), with 10 sessions per mouse collected for analysis (Figures 2, 3, and 4).

For the muscimol experiments, a separate cohort of nine mice was trained initially with P_beep_ = 0.3, then advanced to sessions alternating between P_beep_ = 0.3 and 0.7. P_beep_ = 0 was introduced a day before the manipulation experiment started. One mouse was excluded for lack of sound responsiveness (Figure S1).

For the modified task experiments, a separate cohort of six mice underwent a training schedule similar to the muscimol experimental mice but with a structural manipulation: in the ‘Go’ state, only the first 0.2 s time bin contained a beep, while subsequent bins followed the same stochastic statistics as the ‘Wait’ state. This eliminated the advantage of prolonged waiting. All mice were sound responsive and therefore included in the analysis (Figure S1).

#### Reward rate calculation

The analytical reward rate was computed as in Schwarcz et al.,^15^ with a small adjustment to account for the premature state. For a given waiting time *τ* and P_beep_ (denoted as *θ*), the expected reward rate is given by the closed form expression:

$$r\left(\tau ;\theta \right)=\frac{R\left(\tau ;\theta \right)}{T\left(\tau ;\theta \right)+\left(1-R\left(\tau ;\theta \right)\right)T_{PREM}+T_{ITI}}$$

Here, *R*(*τ*;*θ*) is the probability of obtaining reward when P_beep_ = *θ* and waiting exactly *τ* time units from beep onset; it is given by the analytical expression.

$$R\left(\tau ;\theta \right)=1-\frac{b^{\tau }}{1-c\;\sum_{k=0}^{\tau -1}b^{k}\;}\text{,}$$

where *b*:= (1–*λ*)*θ* is the probability of observing a misleading beep, and *c*:= (1–*λ*)(1–*θ*) is the probability of silence (with *λ* = 0.1). The expression *T*(*τ*;*θ*) in the denominator above is the expected trial duration, defined as the interval between onsets of the wait state:

$$T\left(\tau ;\theta \right)=\frac{\tau b^{\tau }+\sum_{k=0}^{\tau -1}b^{k}\left(\tau \lambda +c\left(k+1\right)\right)}{1-c\sum_{k=0}^{\tau -1}b^{k}}$$

Finally, *T*_*PREM*_ is the premature state duration (7 s), and *T*_*ITI*_ is the average inter-trial interval (3 s).

#### Behavioral data preprocessing and inclusion criteria

Behavioral data were analyzed using MATLAB (MathWorks, USA). Mice were included if their responses exceeded shuffled distributions (Figure S1). For each trial, we preserved the observed choice time (lick latency from trial start) but regenerated a synthetic ‘Wait’ state duration and beep train according to P_beep_. We then recomputed response times (RTs) for the shuffled trials and contrasted the real and shuffled RT distributions. Mice whose real RT distribution did not exceed their shuffled null were excluded from further analyses.

For baseline sessions, we analyzed the first 300 trials per mouse. In muscimol and saline sessions, we restricted analyses to the first 180 trials, as mice tended to stop responding beyond this point following muscimol injection. The same cutoff was applied to saline sessions to ensure comparable trial counts and behavioral engagement across conditions. Because muscimol reduced overall licking (Figure S8), we implemented a rule: if a mouse failed to lick for six consecutive trials, a small water reward was delivered on the next trial to verify continued task engagement. These artificially rewarded trials were excluded from all analyses.

#### Response time definition

Response times (RTs) were defined to avoid bias from stimulus statistics that covary with P_beep_. Because beeps in the ‘Wait’ state become longer and more frequent at higher P_beep_, measuring latency from the onset of individual ‘Wait’ state beeps would artificially shorten RTs at low P_beep_ and inflate differences driven by stimulus statistics rather than waiting behavior. To dissociate waiting from the statistics of the sound stream, we defined RT as the latency from the onset of the sustained beep that subsequently continued into the ‘Go’ state (Figure 2A). Trials were included if they reached this reference-beep onset, regardless of whether the lick occurred during the ‘Go’ state or immediately before it (rewarded and unrewarded-included cases, Figure 2A, top and middle), and excluded if the animal licked before the reference-beep onset was reached (unrewarded-excluded case, Figure 2A, bottom).

#### Responses to local stimulus patterns

We quantified first-lick responses to brief auditory “patterns” embedded in the stimulus stream, computed separately for each mouse and P_beep_. Licking during the ‘Wait’ state interrupts the auditory sequence by triggering a transition to the Premature state mid-time bin, thereby breaking the continuity of the stimulus pattern. We therefore analyzed responses to patterns only in segments where licking does not alter stimulus presentation, specifically, in the Premature timeout and inter-trial interval (ITI) states. For each detected pattern onset, we concatenated licks from the current and subsequent trial to avoid boundary truncation, identified lick onsets using a 0.3 s inter-lick threshold, and took the first lick within 0–7 s as the first lick time; patterns with no lick in this window were counted as “no-lick”. First lick time distributions were binned at 0.1 s and normalized by the total number of detected patterns (including no-lick events, which were treated as probability mass at the upper bound of the window), then averaged across mice. Two pattern sets were analyzed: (i) beep-duration patterns (nominal 200/400/600 ms beeps; Figure 2F), and (ii) matched-silence patterns (three stimuli each containing a 200 ms beep but with 200–600, 400–400, or 600–200 ms of silence before/after the beep; Figure 3B).

To assess how pattern-evoked responses related to behavior across contexts, we compared first lick time distributions for each pattern to reference distributions from single 200 ms beep patterns (Figure 3C). For each mouse and P_beep_ level, we focused on the immediate response by keeping the first 600 ms of the first lick distributions and summing the remaining probability into a seventh bin. The Kullback–Leibler divergence *D*_*KL*_(*p*‖*r*_*c*_) between each pattern distribution *p* and the reference distributions *r*_*c*_ across P_beep_ values was then computed, and each pattern was assigned to the reference with minimal divergence.

#### Behavioral modeling

We modeled response times (RTs) as arising from a mixture of two processes: an evidence accumulation process described by a Wald distribution (inverse Gaussian), and random licking described by an exponential distribution. The mixture proportion corresponded to the probability of lapse responses.

#### Model specification

The probability density of RTs was defined as a mixture of two distributions,

$$p\left(t\left|P_{lapse},\mu ,a,\lambda )=\left(1-P_{lapse}\right)f_{Wald}(t\right|\mu ,a\right)+P_{lapse}f_{\mathrm{Exp}}\left(t|\lambda \right)\text{,}$$

where *t* is the response time, and *P*_*lapse*_∈[0,1] is the lapse probability (mixture weight).

The Wald component was parameterized with drift rate *μ* > 0, decision threshold *a*>0, and unit diffusion (*σ* = 1):

$$f_{Wald}\left(t|\mu ,a\right)=\frac{a}{\sqrt{2\pi t^{3}}}e^{-\frac{{\left(a-\mu t\right)}^{2}}{2t}}$$

This is the first-passage time distribution for a drift–diffusion process with drift μ, threshold a, and unit noise.

The random licking was modeled with an exponential distribution with a rate *λ* > 0,

$$f_{\mathrm{Exp}}\left(t|\lambda \right)=\lambda e^{-\lambda t},for\;t\geq 0$$

Thus, the model has four parameters: *P*_*lapse*_, drift rate *μ*, threshold *a*, and exponential rate *λ*.

#### Censoring

Response times were defined relative to the beep onset after the last potential silence in a trial. If a mouse licked before reaching this reference beep, the resulting value was negative; such trials were excluded, as no positive RT could be defined.

To account for non-lick trials, we treated them as right-censored at 7 s (the duration of the ‘Go’ state). However, simply counting all non-lick trials would inflate the censoring fraction after excluding the negative RTs. We therefore rescaled the number of censored trials so that the overall proportion of no-licks matched that of the full dataset and then filtered the response times. Let *f*_*NL*_ denote the fraction of no-lick trials across all trials (before filtering) and let *n*_*valid*_ be the number of observed RTs with 0 < *t* < 7. The effective number of censored trials included at 7 s was

$$C=\frac{f_{NL}}{1-f_{NL}}n_{valid}\text{,}$$

rounded to the nearest integer. This procedure ensured that the censored count reflected the same no-lick fraction as in the full dataset, while excluding trials with undefined RTs.

#### Fitting procedure

For each animal, global parameter estimates were first obtained by fitting all RTs pooled across P_beep_ conditions. Fits were performed in MATLAB using the Cupid toolbox with censored maximum likelihood estimation (EstMLcensored with bounds [0,7] and censor count C). Optimization used Cupid’s default maximum likelihood routines, with constraints enforcing positive parameters.

This global fit provided initialization values for P_beep_-specific fits. To test how mice adapted their decision strategy to changes in P_beep_, we constructed three models that differed in which parameters were allowed to vary across P_beep_ conditions. This approach allowed us to identify which parameter changes were necessary to explain the observed context dependence of behavior:

**Table:** 

Model	*P*_*lapse*_ (mixture weight)	*μ* (drift rate)	*a* (threshold)	*λ* (exponential rate)
Full	**Varies**	**Varies**	Fixed	**Varies**
Timing	Fixed	**Varies**	Fixed	**Varies**
Probabilistic	**Varies**	Fixed	Fixed	**Varies**

“Varies” = fit separately per P_beep_; “Fixed” = held at the global fit.

Fixing *a* across models avoids drift–threshold trade-offs and allows us to isolate changes in accumulation speed.

#### Model evaluation

Goodness of fit was assessed by *R*^2^ (defined as $1-\frac{SSE}{SST}$) between the first 3 s of mean empirical and model-predicted RT distributions (Figures 4, 5, and 6). Formal model comparison used Akaike Information Criterion (AIC) with group-level statistics computed on AIC differences across animals.

### Quantification and statistical analysis

#### Statistical analysis

All statistical analyses were performed using MATLAB (MathWorks, USA; version 2023b). Statistical details for each analysis, including the statistical test used, exact *p* values, sample size, and definition of n, are reported in the Results, figures, and figure legends. Unless otherwise stated, n refers to the number of mice. The baseline behavioral dataset included 15 mice, the OFC inactivation dataset included 8 mice, and the modified-task dataset included 6 mice.

Linear mixed-effects models (LME) were used to account for repeated measures across animals, with mouse identity included as a random effect. For interaction LMEs, continuous predictors (e.g., P_beep_, beep duration, preceding silence) were mean-centered prior to fitting, and interactions were specified on the centered terms. Within-animal comparisons (e.g., high vs. low P_beep_; timing vs. probabilistic model; saline vs. muscimol; baseline vs. modified task) were assessed with paired t-tests. Pearson’s correlation coefficients were calculated to assess relationships between trial-by-trial response times and P_beep_. We then performed group-level one-sample t-tests at each trial offset relative to the block switch and corrected for multiple comparisons using false discovery rate (FDR; Benjamini-Hochberg). All reported error bars denote the standard error of the mean (SEM) across animals unless otherwise specified. Statistical significance was set at *p* < 0.05. *p*-values smaller than 10^−6^ are reported as *p* < 10^−6^.

Animals were excluded from analysis if they did not show sound-responsive behavior, as defined by comparison of real response-time distributions to shuffled null distributions. Specifically, 2 of 17 mice were excluded from the baseline behavioral cohort, and 1 of 9 mice was excluded from the OFC inactivation cohort. No mice were excluded from the modified-task cohort. Artificially rewarded trials delivered after six consecutive no-lick trials were excluded from all analyses. Trials with undefined response times were excluded from response-time analyses, and no-lick trials were treated as right-censored observations for model fitting, as described in Method Details.

##### Parameter visualization

For fitted-parameter plots, we applied a display-only filter: within each context, points exceeding the across-mouse mean +3 SD were omitted from the plot. All LMEs and model comparisons used the full, unfiltered data. Summary curves show the median across mice.

## Supplemental information

Document S1. Figures S1–S9

Table S1. Statistical information for Figures 2–6 Summary of the statistical tests, sample sizes, model terms, effect estimates, and p values reported for the main figures.
