# Supplementary material for: Orbitofrontal cortex contributes to context-dependent timing under ambiguity
Source: iScience. 2026 Aug 10;29(8):117110. doi: 10.1016/j.isci.2026.117110 (PMC13486877; doi:10.1016/j.isci.2026.117110)
Supplement: Supplementary file 1 [file mmc1.pdf]

iScience, Volume *29*

Supplemental information

Orbitofrontal cortex contributes to context-dependent timing under ambiguity

Gabrielle Marmur, Haneen Rajabi, John Schwarcz, Robert Reiner, Jonathan Kadmon, and Eran Lottem

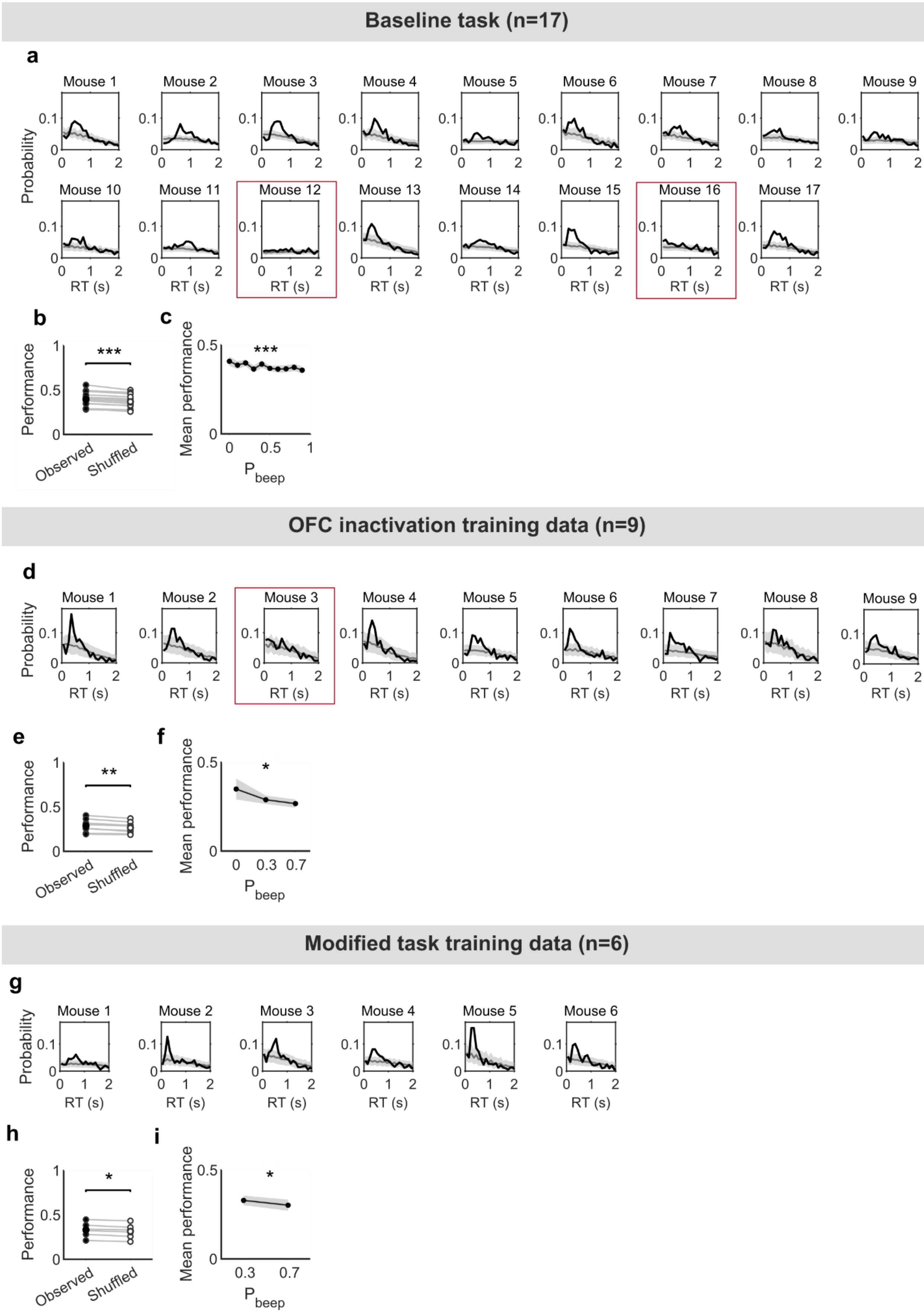
Baseline task (n=17)
a
Mouse 1
Mouse 2
Mouse 3
Mouse 4
Mouse 5
Mouse 6
Mouse 7
Mouse 8
Mouse 9
Mouse 10
Mouse 11
Mouse 12
Mouse 13
Mouse 14
Mouse 15
Mouse 16
Mouse 17
Probability
0.1
0
0 1 2
RT (s)
b
Performance
1
0.5
0
***
Observed
Shuffled
c
Mean performance
0.5
0
***
0 0.5 1
P beep
OFC inactivation training data (n=9)
d
Mouse 1
Mouse 2
Mouse 3
Mouse 4
Mouse 5
Mouse 6
Mouse 7
Mouse 8
Mouse 9
Probability
0.1
0
0 1 2
RT (s)
e
Performance
1
0.5
0
**
Observed
Shuffled
f
Mean performance
0.5
0
*
0 0.3 0.7
P beep
Modified task training data (n=6)
g
Mouse 1
Mouse 2
Mouse 3
Mouse 4
Mouse 5
Mouse 6
Probability
0.1
0
0 1 2
RT (s)
h
Performance
1
0.5
0
*
Observed
Shuffled
i
Mean performance
0.5
0
*
0.3 0.7
P beep

Figure S1. Behavioral responsiveness and performance across all datasets. a–c, Baseline task (batch 1). a, Response-time (RT) distributions (black) for individual mice (n = 17) compared with the mean (dark grey) and 5–95% envelope (light grey shading) of shuffled RT distributions generated from the same trial structure. Mice were considered responsive if their RT distribution exhibited a clear peak exceeding the shuffled envelope, indicating stimulus-locked licking behavior; non-responsive mice (red frames) were excluded from further analyses. b, Percent correct (fraction of rewarded trials) for observed data compared to a shuffled baseline (within-mouse reassignment of choice times). Mice performed significantly better than shuffled (paired t-test, $p = 6.30 \times 10^{-7}$, n=15 mice). c, Percent correct as a function of P_{beep} for included mice (mean ± SEM across mice; n=15 mice). A linear mixed-effects model revealed a significant effect of P_{beep} ($p = 1.11 \times 10^{-4}$).

d–f, OFC inactivation cohort (training sessions preceding manipulation; n = 9). d, Same analysis as in (a), used to identify responsive mice (one excluded). e, Observed percent correct exceeds shuffled baseline (paired t-test, $p = 1.04 \times 10^{-3}$, n=8 mice). f, Percent correct as a function of P_{beep}, showing a modest but significant effect (linear mixed-effects model, p = 0.032).

g–i, Modified-task cohort (n = 6). g, Same responsiveness analysis as in (a); all mice were included. h, Observed percent correct exceeds shuffled baseline (paired t-test, $p = 1.42 \times 10^{-3}$). i, Percent correct as a function of P_{beep} (mean ± SEM across mice; n=6 mice), with a significant effect of P_{beep} (linear mixed-effects model, p = 0.020).

Across all datasets, mice perform above a shuffled baseline, confirming stimulus-dependent behavior. However, percent correct provides only a coarse measure of performance in this task, as relatively high reward rates can be achieved even with weak stimulus alignment due to the long response window. For this reason, subsequent analyses focus on response-time distributions and their context-dependent modulation, which more directly capture stimulus-guided behavior.

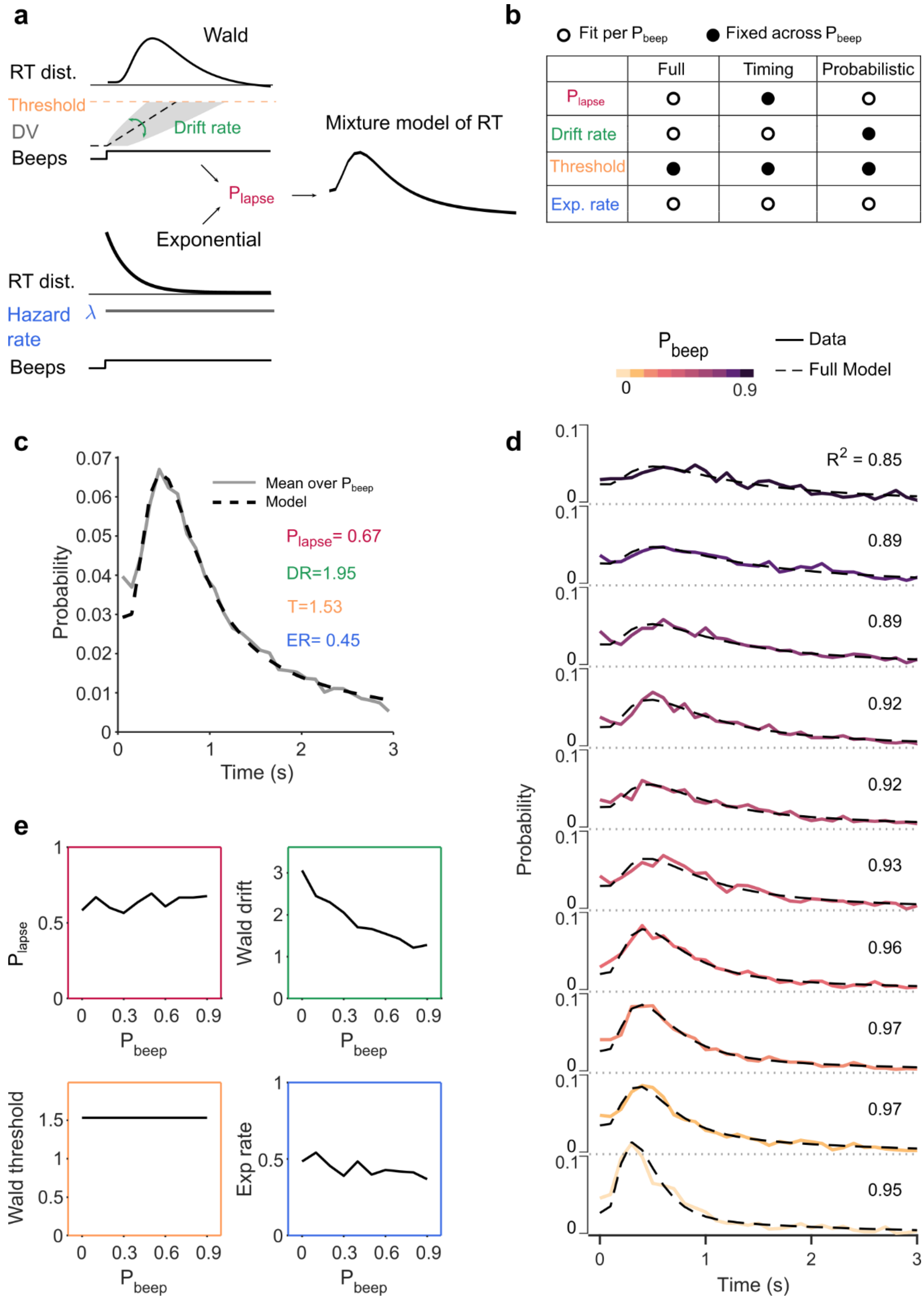
a
Wald
RT dist.
Threshold
DV
Drift rate
Beeps
P_lapse
Mixture model of RT
Exponential
RT dist.
Hazard rate
λ
Beeps
b
Fit per P_beep
Fixed across P_beep
Full
Timing
Probabilistic
P_lapse
Drift rate
Threshold
Exp. rate
P_beep
0
0.9
Data
Full Model
c
Probability
Time (s)
Mean over P_beep
Model
P_lapse= 0.67
DR=1.95
T=1.53
ER= 0.45
d
R^2 = 0.85
0.89
0.89
0.92
0.92
0.93
0.96
0.97
0.97
0.95
Probability
Time (s)
e
P_lapse
Wald drift
Wald threshold
Exp rate
P_beep

Figure S2. Mixture model fitting procedure and parameter structure.

a, Schematic of the response-time (RT) mixture model combining a Wald (drift-diffusion-like) process and an exponential "lapse" process. Four model parameters are color-coded: lapse probability (P_{lapse}, red), Wald drift rate (green), Wald threshold (orange), and exponential rate (blue). **b,** Parameter structure for the three model variants shown in the main text (Full, Timing, and Probabilistic). Open circles indicate parameters fit separately for each P_{beep} condition, whereas filled circles indicate parameters fixed across P_{beep}. **c–e,** Example of the fitting procedure shown for the RT distribution of pooled data across the 15 mice in batch 1. **c,** First step of the fitting procedure: the mean RT distribution (averaged over all P_{beep} conditions) is fit to estimate the parameters designated as "fixed." Example data (black) and model fit (dashed line) are shown with the resulting best-fit parameter values. **d,** Second step: using the fixed parameters obtained from the mean fit, the model is refit separately to RT distributions from each P_{beep} condition, adjusting only the parameters allowed to vary. Colored solid lines show the empirical distributions, and black dashed lines show the model fits. Goodness-of-fit (R^2) values are shown for each condition. **e,** Fitted parameter values as a function of P_{beep}. In this example, the full model was used: the Wald threshold was fixed across P_{beep}, while the other parameters (P_{lapse}, drift rate, and exponential rate) were fit independently for each context.

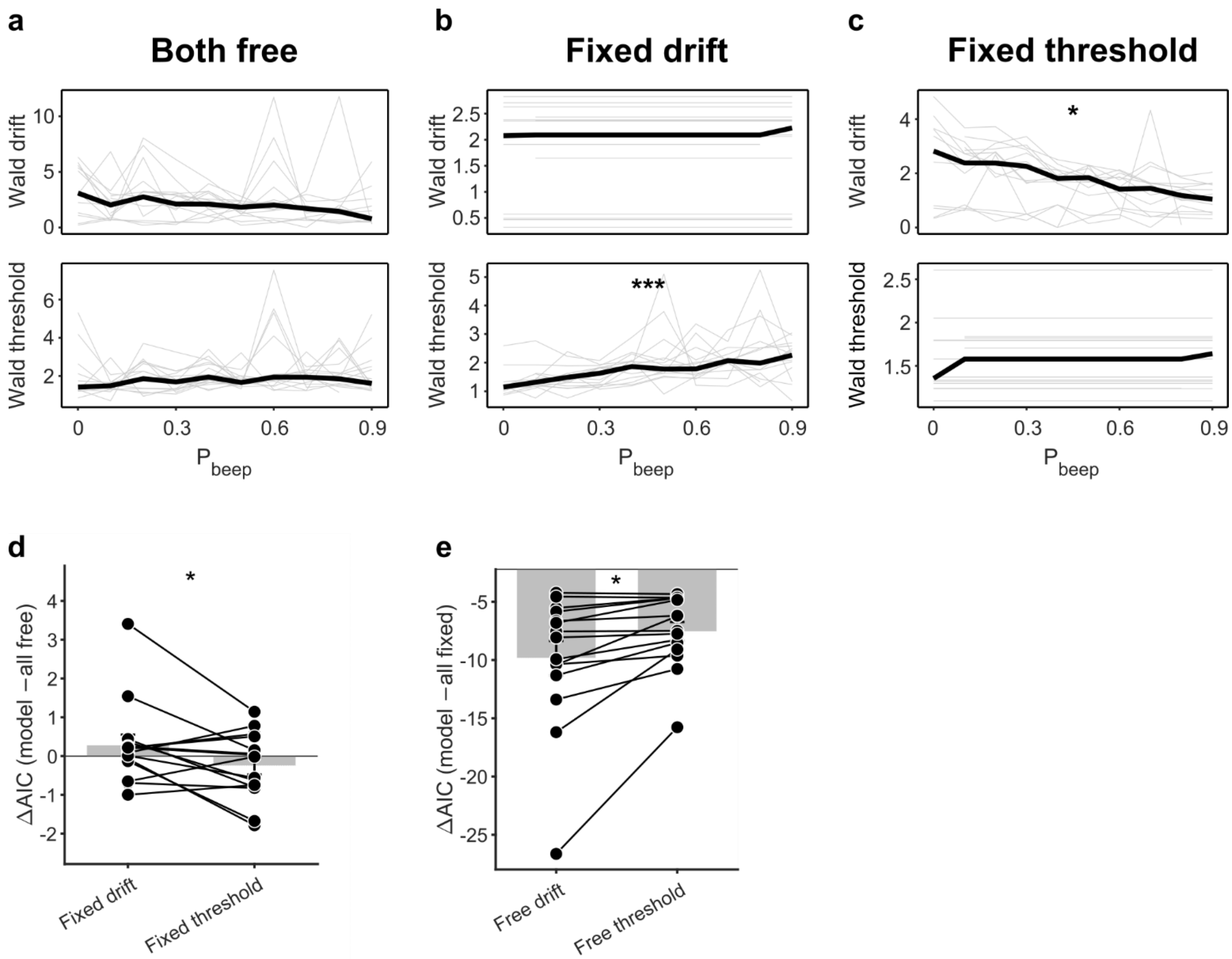

Figure S3. Drift rate and threshold are interdependent, but allowing drift to vary with P_{beep} better captures behavioral adaptation.

The mixture model includes two interacting components: a random-licker process (one parameter) and an accumulator process (two parameters: drift rate and threshold). Because both drift and threshold shape the accumulator's response-time distribution, changes in one can offset changes in the other, leading to interdependence. **a–c,** Fitted parameter estimates for the 15 mice in the baseline cohort illustrate this relationship. Thin grey lines show individual mice and thick black lines show the population median. When both parameters are fit freely (a), both show the expected trend across P_{beep} but neither is individually significant. Fixing drift reveals a significant effect of P_{beep} on threshold (b, linear mixed-effects model, $p < 0.001$), whereas fixing threshold reveals a significant effect for drift (c, linear mixed-effects model, $p = 0.0156$). **d–e,** Model comparisons (ΔAIC relative to reference models) indicate that variation in drift explains the data better: fixing drift worsens the fit compared to fixing threshold (d, two-tailed paired t-test, $p = 0.0441$), while

freeing drift improves the fit more than freeing threshold (e, two-tailed paired t-test, $p = 0.0110$). These results suggest that drift and threshold jointly capture a single underlying timing process; we fix the threshold in subsequent analyses to isolate this process and quantify its modulation by P_{beep}.

n=15 mice. $*p<0.05$, $***p<0.001$.

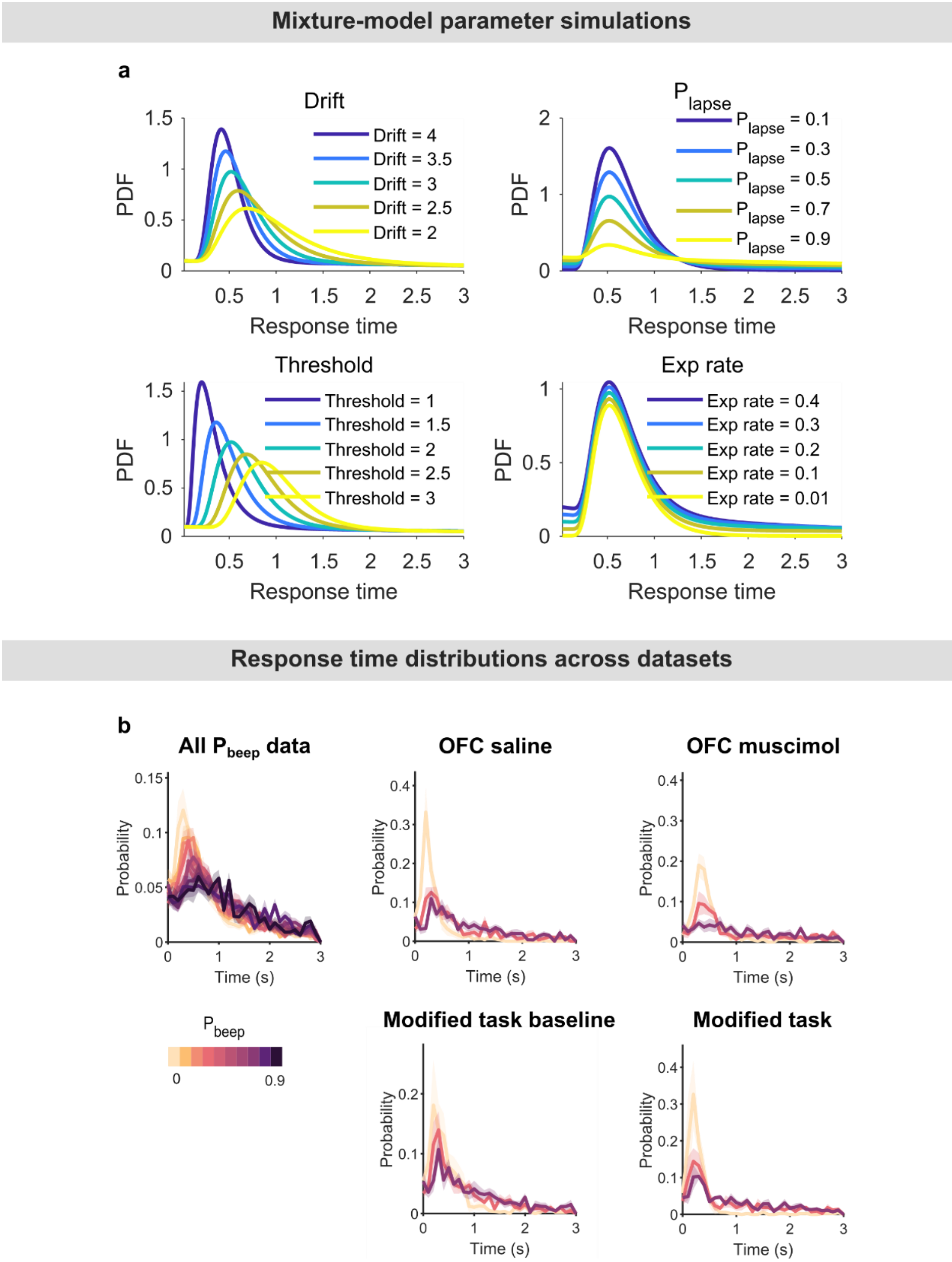

Figure S4. Simulations illustrate distinct effects of mixture-model parameters on response-time distributions.

a, Simulated response-time distributions generated by selectively varying individual parameters of the Wald + exponential mixture model while holding all other parameters fixed. Increasing drift rate shifted the timing of the accumulation-driven peak toward shorter response times. Increasing the threshold shifted the entire distribution toward

longer response times. In contrast, increasing lapse probability (P_{lapse}) primarily reduced the relative contribution of the peaked accumulation component while increasing the diffuse exponential component, with comparatively little effect on peak timing. Changes in the exponential rate produced a global change in the distribution, increasing or decreasing the overall probability of licking without shifting the timing of the accumulation-driven peak or the relative contribution of the accumulation process.

b, Mean response-time distributions across P_{beep} for the different experimental conditions. Lines and shading show mean ± SEM across mice (baseline task, n=15; OFC saline and muscimol, n=8; modified-task baseline and modified task, n=6). Changes across contexts were characterized primarily by shifts in peak timing, qualitatively resembling the effects of drift modulation shown in (a). In contrast, under OFC inactivation and in the modified task, distributions remained more temporally aligned across P_{beep} while varying in the relative weight of early diffuse responses, qualitatively resembling modulation of P_{lapse}. Together, these comparisons provide intuition for how different model parameters contribute to the distinct patterns of context-dependent adaptation observed across datasets.

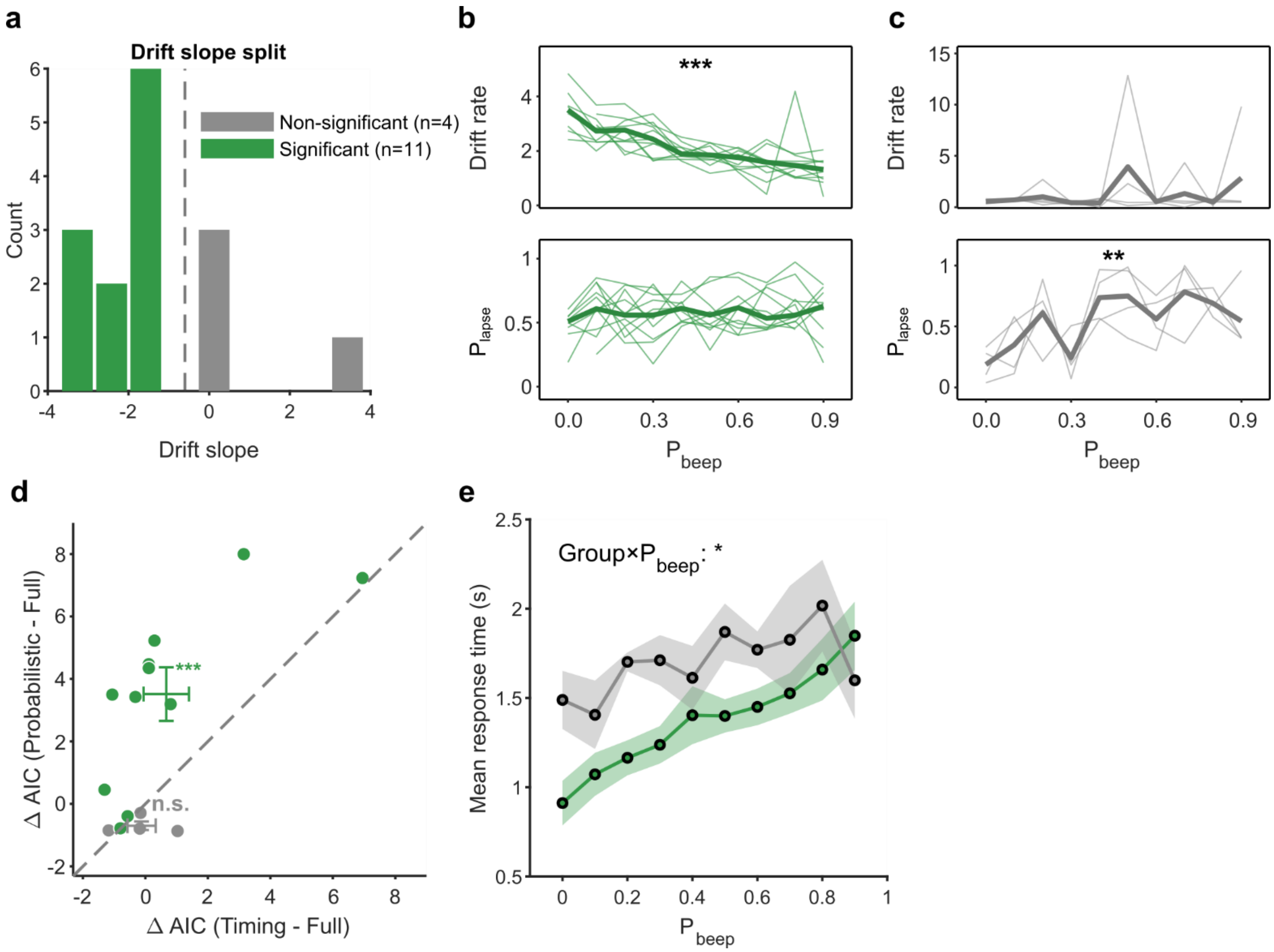

Figure S5. Drift adaptation is evident in most mice, while a minority show no significant modulation.

a, Across the 15 mice in the baseline cohort, robust regression of Wald drift rate against P_{beep} classified 11 mice as "drift adapters", with significant negative drift-P_{beep} slopes (green), and 4 mice as non-adapters, with non-significant slopes (grey). **b-c,** Fitted parameters for drift adapters (n=11; b) and non-adapters (n=4; c). Thin lines show individual mice and thick lines show the group median. In drift adapters, drift rate decreased significantly with P_{beep} (linear mixed-effects model, $p = 2.4 \times 10^{-21}$), whereas P_{lapse} showed no significant modulation ($p = 0.14$). In non-adapters, drift rate did not vary significantly with P_{beep} ($p = 0.27$), whereas P_{lapse} increased significantly ($p = 0.0027$). **d,** Model-comparison analysis showing ΔAIC between the Timing and Probabilistic model variants (relative to the Full model). Points above the identity line indicate a better fit for the Timing model. Drift adapters were significantly better fit by the Timing model (paired

t-test, $p = 7.7 \times 10^{-4}$, n=11 mice), while non-adapters showed no significant difference ($p = 0.31$, n=4 mice). **e,** Mean response times as a function of P_{beep} and group. Points and shading show mean ± SEM across mice (n=11 drift adapters; n=4 non-adapters). A significant Group × P_{beep} interaction was found using a linear mixed-effects model ($p = 0.010$), confirming distinct behavioral adaptation profiles between drift adapters and non-adapters.

$*p<0.05$, $**p<0.01$, $***p<0.001$; n.s., not significant.

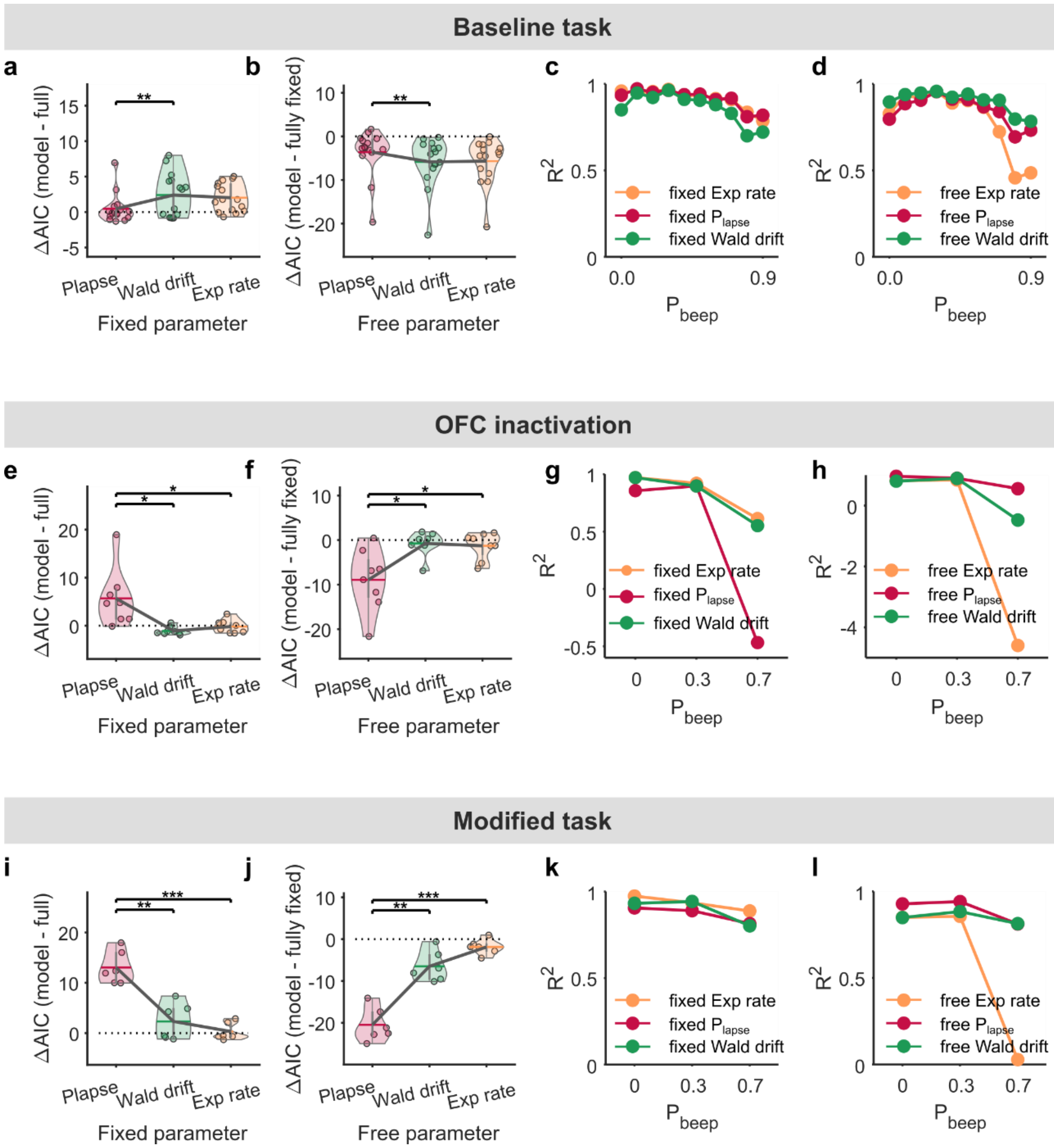

Figure S6. Parameter contributions to context-dependent adaptation differ across datasets.

The full mixture model includes three parameters that may vary with P_{beep}: Wald drift rate, lapse probability (P_{lapse}), and exponential rate. Model variants were constructed by fixing or freeing individual parameters across P_{beep}, and evaluated using ΔAIC (relative to the full model) and coefficient of determination ($R^2 = 1 - \frac{SSE}{SST}$). R^2 was calculated between the group-mean empirical and model-predicted RT distributions for each P_{beep} condition.

a–d, **Baseline task.** Fixing drift rate produced the largest increase in AIC, significantly worse than fixing P_{lapse} (paired t-test, $p = 0.0064$) but not different from fixing exponential rate ($p = 0.50$; n=15 mice). Allowing drift to vary yielded the strongest improvement in fit, outperforming P_{lapse} ($p = 0.0015$) but not exponential rate ($p = 0.82$; paired t-tests, n=15

mice). R^2 analyses showed the same pattern: fixing drift resulted in the lowest R^2 values, whereas allowing drift to vary produced the highest. Repeated-measures ANOVAs across the ten P_{beep} conditions confirmed significant model effects: for fixed-parameter models, $F(2,18) = 14.24$, $p = 0.0002$, with the fixed-drift model significantly lower than both fixed-lapse ($p = 0.0056$) and fixed-exponential ($p = 0.016$); for free-parameter models, $F(2,18) = 5.70$, $p = 0.012$, with the free-drift model significantly higher than both free-lapse ($p = 0.0010$) and free-exponential ($p = 0.027$). Together, these results indicate that drift modulation best captures context-dependent adaptation in the baseline task.

e–h, **OFC inactivation.** Fixing P_{lapse} produced the largest increase in AIC, significantly worse than fixing drift ($p = 0.019$) or exponential rate ($p = 0.044$; paired t-tests, n=8 mice). Allowing P_{lapse} to vary yielded the strongest improvement in fit, outperforming both drift ($p = 0.014$) and exponential rate ($p = 0.021$; paired t-tests, n=8 mice). R^2 analyses mirrored this pattern, with lowest fits when P_{lapse} was fixed and highest fits when it was allowed to vary. These results indicate that, following OFC inactivation, behavioral adaptation is primarily captured by modulation of lapse probability.

i–l, **Modified task.** Fixing P_{lapse} again produced the largest increase in AIC, significantly worse than fixing drift ($p = 0.0084$) or exponential rate ($p = 0.00079$; paired t-tests, n=6 mice). Allowing P_{lapse} to vary yielded the strongest improvement in fit, outperforming both drift ($p = 0.0023$) and exponential rate ($p = 0.00052$; paired t-tests, n=6 mice). R^2 analyses showed the same pattern, with lowest fits when P_{lapse} was fixed and highest fits when it was allowed to vary. Thus, under the modified task, adaptation is best explained by variation in lapse probability.

Across datasets, both ΔAIC and R^2 analyses consistently identify the same parameter as critical: drift in the baseline task, and lapse probability under OFC inactivation and in the modified task.

$*p<0.05$, $**p<0.01$, $***p<0.001$.

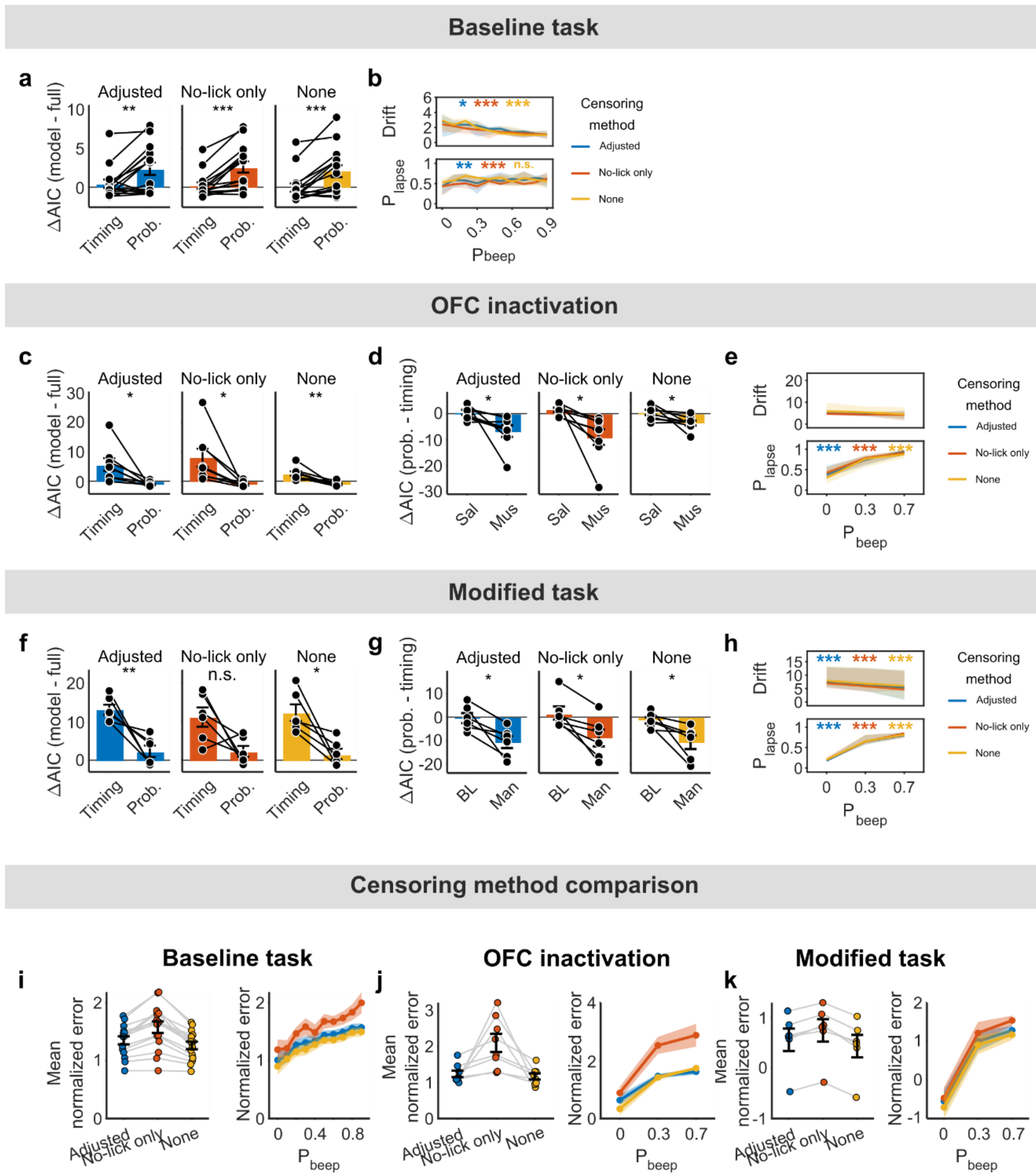

Figure S7. Robustness of model comparison and parameter estimates to censoring methods.

All analyses were performed using three censoring approaches: no censoring ("None"), censoring only no-lick trials ("No-lick only"), and the adjusted censoring procedure used in the main text ("Adjusted"). Across all methods, the main qualitative results were preserved. Baseline task, n=15 mice; OFC inactivation, n=8 mice; modified task, n=6 mice.

a, c, f, Model comparison between the timing (drift-based) and probabilistic (lapse-based) variants of the mixture model (ΔAIC relative to the full model). In the baseline task (a), the timing model provided a better fit than the probabilistic model (paired t-test). In

contrast, under OFC inactivation (c) and in the modified task (f), the probabilistic model provided a better fit across all censoring methods (paired t-tests).

b, e, h, Parameter estimates as a function of P_{beep} for each censoring method. In the baseline task (b), drift rate varied systematically with P_{beep}, while lapse probability showed weaker or no consistent modulation (linear mixed-effects models). Under OFC inactivation (e) and in the modified task (h), drift modulation was reduced, whereas lapse probability increased significantly with P_{beep} across censoring methods.

d, g, Direct comparison of model preference across conditions ($\Delta AIC_{probabilistic - timing}$). Positive values indicate a better fit of the timing model, whereas negative values indicate a better fit of the probabilistic model. In both OFC inactivation (d) and the modified task (g), the relative preference for the probabilistic model was significantly greater than in the corresponding baseline condition (paired t-tests), and this pattern was consistent across censoring methods.

i–k, Comparison of model fit quality across censoring methods using mean normalized error (model error divided by the number of included trials). Left panels show per-mouse averages; right panels show error as a function of P_{beep}. Across datasets, a Friedman test revealed a significant effect of censoring method (baseline: $p = 3.06 \times 10^{-7}$; OFC inactivation: $p = 8.05 \times 10^{-4}$; modified task: $p = 0.0025$). Pairwise comparisons showed that the no-censoring approach yielded the lowest error across methods, indicating improved numerical fit. However, this approach excludes no-lick trials and therefore does not capture the full distribution of behavioral outcomes. Among the censoring-based approaches, the adjusted method consistently provided better fits than the simpler "no-lick only" procedure. Despite differences in fit quality, all key qualitative effects were preserved across methods.

Taken together, these analyses demonstrate that the main conclusions are robust to the choice of censoring procedure. While simpler approaches can yield marginally improved fits, the adjusted censoring method provides a principled way to incorporate no-lick outcomes and was therefore used in the main analyses.

$*p<0.05$, $**p<0.01$, $***p<0.001$; n.s., not significant.

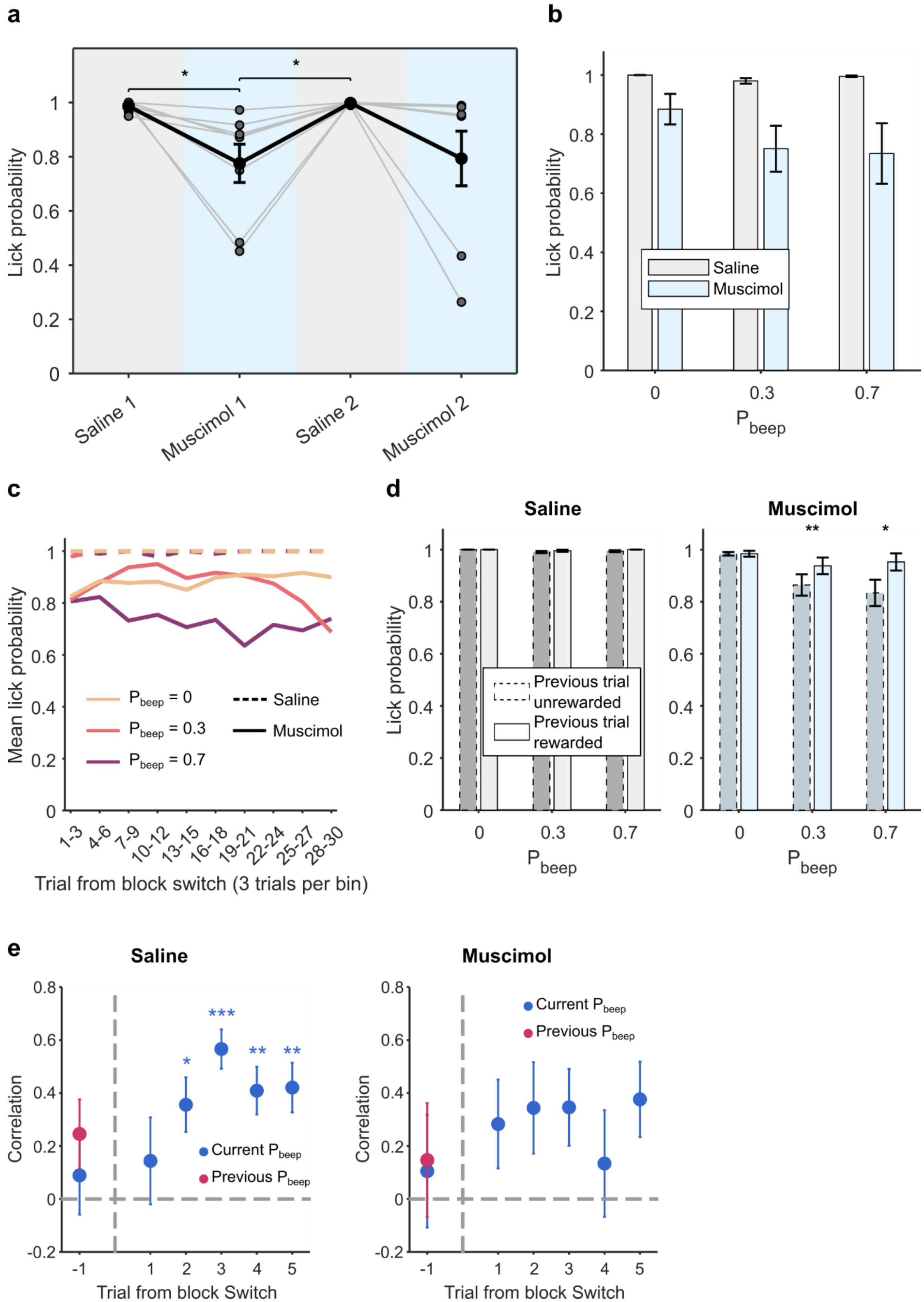
a
b
*
*
Lick probability
1
0.8
0.6
0.4
0.2
0
Saline 1
Muscimol 1
Saline 2
Muscimol 2
Lick probability
1
0.8
0.6
0.4
0.2
0
Saline
Muscimol
0
0.3
0.7
P_{beep}
c
d
Mean lick probability
1
0.8
0.6
0.4
0.2
0
P_{beep} = 0
P_{beep} = 0.3
P_{beep} = 0.7
Saline
Muscimol
1-3
4-6
7-9
10-12
13-15
16-18
19-21
22-24
25-27
28-30
Trial from block switch (3 trials per bin)
Saline
Muscimol
Lick probability
1
0.8
0.6
0.4
0.2
0
Previous trial unrewarded
Previous trial rewarded
**
*
0
0.3
0.7
P_{beep}
0
0.3
0.7
P_{beep}
e
Saline
Muscimol
Correlation
0.8
0.6
0.4
0.2
0
-0.2
*
***
**
**
Current P_{beep}
Previous P_{beep}
-1
1
2
3
4
5
Trial from block Switch
Correlation
0.8
0.6
0.4
0.2
0
-0.2
Current P_{beep}
Previous P_{beep}
-1
1
2
3
4
5
Trial from block Switch

Figure S8. OFC inactivation causes a context-dependent reduction in lick probability.

All panels show data from n=8 mice.

a, Lick probability for each mouse across the four inactivation-week sessions. Lick probability was defined as the fraction of trials in which the mouse emitted at least one lick before the end of the response window. Muscimol suppressed licking on the first inactivation day, with recovery on the subsequent saline session ($p = 0.035$ for both transitions, FDR-corrected). A reduction on the second muscimol session was observed but was not significant ($p = 0.080$). Black markers indicate mean ± SEM.

b, Lick probability across P_{beep} conditions (0, 0.3, 0.7) for saline and muscimol sessions. Bars represent mean ± SEM across mice. A linear mixed-effects model revealed a significant main effect of injection substance ($p = 0.022$) and P_{beep} ($p = 0.030$), but no significant interaction ($p = 0.126$). Within-mouse comparisons (saline − muscimol) showed weaker suppression at $P_{beep} = 0$ relative to $P_{beep} = 0.7$ ($p = 0.044$), with a similar trend at $P_{beep} = 0.3$ ($p = 0.058$).

c, Within-block dynamics of lick probability, computed in non-overlapping bins of three trials after each block switch. Lines show mean lick probability across mice. Under muscimol, lick probability decreased across the block in high-P_{beep} contexts, but slightly increased in $P_{beep} = 0$ blocks. A linear mixed-effects model revealed a significant P_{beep} × Trial bin interaction ($p = 0.0034$) and a three-way P_{beep} × Injection Substance × Trial bin interaction ($p = 0.031$), indicating that context-dependent within-block changes were specific to muscimol sessions.

d, Lick probability as a function of P_{beep}, split by previous trial outcome (unrewarded vs. rewarded; analysis restricted to trials following a lick). Under saline (left), previous outcome had no detectable effect. Under muscimol (right), mice licked less following an unrewarded trial, specifically at higher P_{beep} values ($P_{beep} = 0.3$: $p_{FDR} = 0.0064$; $P_{beep} = 0.7$: $p_{FDR} = 0.0425$; $P_{beep} = 0$: $p_{FDR} = 1$). Bars represent mean ± SEM across mice (n = 8).

e, Correlation between response times and P_{beep} as a function of trial position relative to block switches, shown separately for saline (left) and muscimol (right) sessions. Points show mean ± SEM across mice. Correlations were computed within each mouse and tested against zero across animals using one-sample t-tests with FDR correction. Under saline, response times rapidly become correlated with the current P_{beep} following a block switch, indicating fast adaptation to the new context. Under muscimol, this correlation is reduced and develops more slowly, consistent with impaired updating of response timing following context changes.

∗$p<0.05$, ∗∗$p<0.01$, ∗∗∗$p<0.001$, following FDR correction.

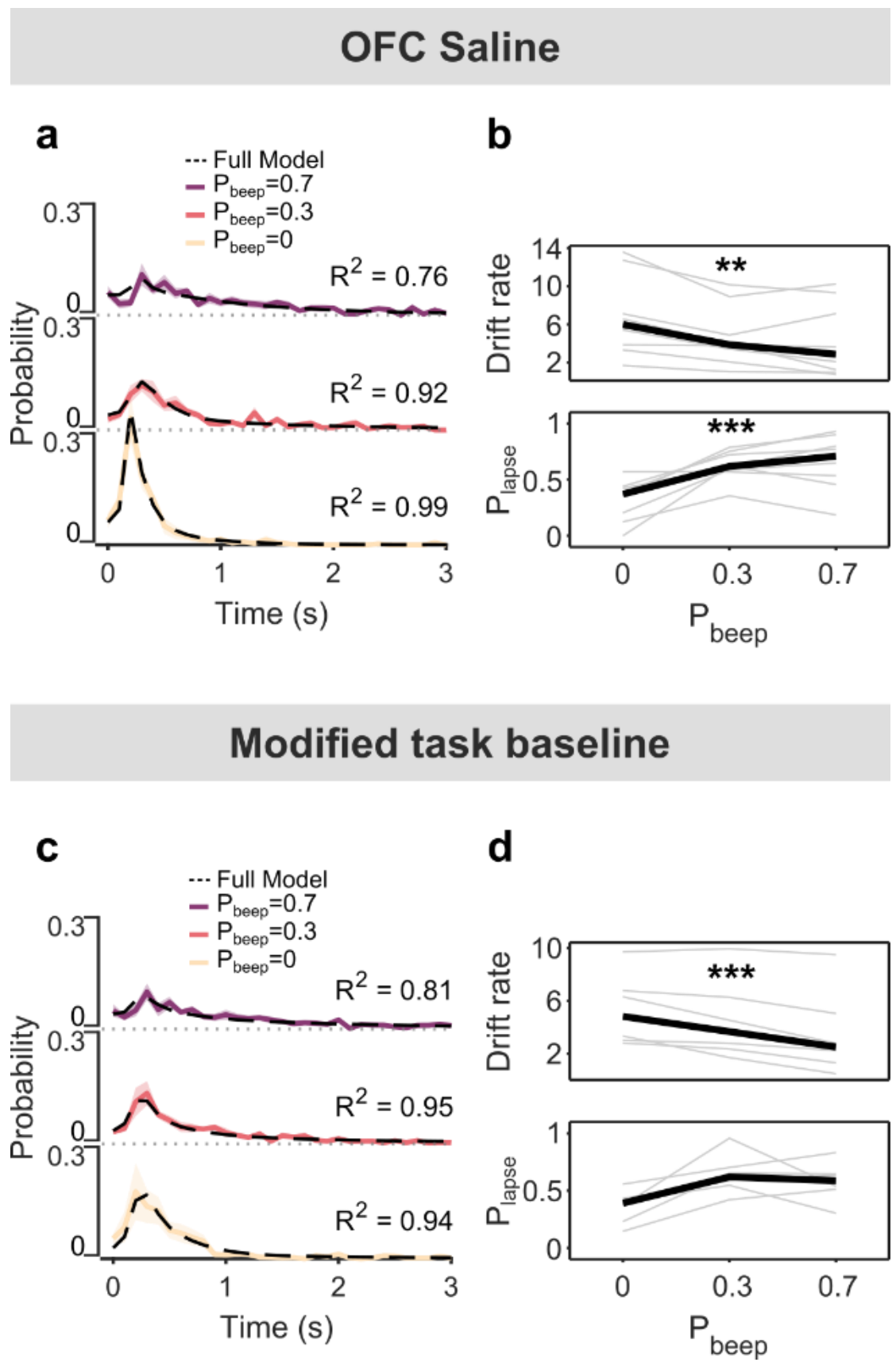

Figure S9. Baseline-condition model fits for the OFC saline and modified-task cohorts.

a, Fits of the full mixture model to response-time distributions across P_{beep} for the saline sessions of the OFC inactivation cohort (0.1 s bins, mean ± SEM across n=8 mice). Colored lines show empirical distributions and dashed black lines show model fits. Distribution peaks shifted systematically across contexts, consistent with context-dependent changes in response timing.

b, Corresponding parameter estimates across P_{beep}. Both Wald drift rate (top; linear mixed-effects model, $p = 0.0012$) and lapse probability (P_{lapse}, bottom; $p = 3.58 \times 10^{-4}$) varied significantly with P_{beep} (n=8 mice). Thin grey lines show individual mice; thick black lines show the population median.

c, Fits of the full mixture model to response-time distributions across P_{beep} for the baseline sessions of the modified-task cohort (0.1 s bins, mean ± SEM across n=6 mice). Colored lines show empirical distributions and dashed black lines show model fits. As in the original task, response-time distributions shifted across contexts.

d, Corresponding parameter estimates across P_{beep}. Drift rate varied significantly with P_{beep} (linear mixed-effects model, $p = 7.28 \times 10^{-5}$), whereas P_{lapse} did not ($p = 0.054$; n=6 mice). Thin grey lines show individual mice; thick black lines show the population median.
